# Virgin Olive Oil Phenolic Compounds Modulate the HDL Lipidome in Hypercholesterolaemic Subjects: A Lipidomic Analysis of the VOHF Study

**DOI:** 10.1002/mnfr.202001192

**Published:** 2021-03-18

**Authors:** Sara Fernández‐Castillejo, Anna Pedret, Úrsula Catalán, Rosa‐Maria Valls, Marta Farràs, Laura Rubió, Olga Castañer, Alba Macià, Montse Fitó, Maria José Motilva, Maria‐Isabel Covas, Martin Giera, Alan T. Remaley, Rosa Solà

**Affiliations:** ^1^ Facultat de Medicina i Ciències de la Salut, Departament de Medicina i Cirurgia, Universitat Rovira i Virgili, Grup Nutrició Funcional, Oxidació i Malalties Cardiovasculars (NFOC‐Salut) Reus 43201 Spain; ^2^ Eurecat, Centre Tecnològic de Catalunya Unitat de Nutrició i Salut Reus 43204 Spain; ^3^ Institut de Recerca de l'Hospital Santa Creu i Sant Pau‐Institut d'Investigacions Biomèdiques (IIB) Sant Pau Barcelona 08041 Spain; ^4^ CIBER de Diabetes y Enfermedades Metabólicas Asociadas (CIBERDEM) Instituto de Salud Carlos III Madrid 28029 Spain; ^5^ Food Technology Department, XaRTA‐TPV, Agrotecnio Center, Escola Tècnica Superior d'Enginyeria Agrària University of Lleida. Avda/ Alcalde Rovira Roure 191 Catalonia Lleida 25198 Spain; ^6^ Cardiovascular Risk and Nutrition Research Group Hospital del Mar Medical Research Institute (IMIM) Barcelona 08003 Spain; ^7^ PhD Program in Biomedicine Universitat Pompeu Fabra Barcelona 08005 Spain; ^8^ Consorcio CIBER, M.P. Fisiopatología de la Obesidad y Nutrición (CIBEROBN) Instituto de Salud Carlos III Madrid 28029 Spain; ^9^ Instituto de Ciencias de la Vid y del Vino‐ICVV (Consejo Superior de Investigaciones Científicas‐CSIC Universidad de La Rioja, Gobierno de La Rioja), Finca La Grajera, Ctra. de Burgos Km. 6 (LO‐20 ‐ salida 13) Logroño (La Rioja) 26007 Spain; ^10^ NUPROAS Handelsbolag (NUPROAS HB) Nacka Sweden; ^11^ Center for Proteomics and Metabolomics Leiden University Medical Center Albinusdreef 2 Leiden 2333ZA The Netherlands; ^12^ Department of Laboratory Medicine Clinical Center National Institutes of Health Bethesda MD 20814 USA; ^13^ Lipoprotein Metabolism Section Cardio‐Pulmonary Branch National Heart Lung and Blood Institute National Institutes of Health Bethesda MD 20814 USA; ^14^ Institut d'Investigació Sanitaria Pere Virgili (IISPV) Reus 43204 Spain; ^15^ Hospital Universitari Sant Joan de Reus Reus 43204 Spain

**Keywords:** cholesterol efflux, HDL functionality, lipidomics, polyphenols, resistance to oxidation

## Abstract

**Scope:**

The lipidomic analysis of high‐density lipoprotein (HDL) could be useful to identify new biomarkers of HDL function.

**Methods and results:**

A randomized, controlled, double‐blind, crossover trial (33 hypercholesterolaemic subjects) is performed with a control virgin olive oil (VOO), VOO enriched with its own phenolic compounds (FVOO), or VOO enriched with additional phenolic compounds from thyme (FVOOT) for 3 weeks. HDL lipidomic analyses are performed using the Lipidyzer platform. VOO and FVOO intake increase monounsaturated‐fatty acids (FAs) and decrease saturated and polyunsaturated FAs in triacylglyceride (TAG) species, among others species. In contrast, FVOOT intake does not induce these FAs changes. The decrease in TAG52:3(FA16:0) after VOO intake and the decrease in TAG52:5(FA18:2) after FVOO intake are inversely associated with changes in HDL resistance to oxidation. After FVOO intake, the decrease in TAG54:6(FA18:2) in HDL is inversely associated with changes in HDL cholesterol efflux capacity.

**Conclusion:**

VOO and FVOO consumption has an impact on the HDL lipidome, in particular TAG species. Although TAGs are minor components of HDL mass, the observed changes in TAG modulated HDL functionality towards a cardioprotective mode. The assessment of the HDL lipidome is a valuable approach to identify and characterize new biomarkers of HDL function.

## Graphical abstract

The sustained intake of virgin olive oil (VOO) and a functional VOO enrich with its own phenolic compounds (FVOO) modify the fatty acid profile in the high‐density lipoprotein (HDL) triacylglycerides, among others, whereas a functional VOO enrich with phenolic compounds from thyme (FVOOT) does not induce these changes. These changes are associated with changes in HDL functionality.

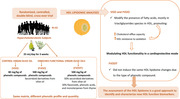

## 1 Introduction

Based on multiple intervention and gene association studies, high‐density lipoprotein (HDL) cholesterol (HDL‐C) is now recognized as an imperfect measure of cardiovascular disease (CVD) risk, suggesting that emerging HDL‐related parameters may be useful for the clinical management of patients with CVD.^[^ ^1^ ^]^ In particular, HDL functional quality seems to be a more important biomarker than HDL‐C levels.^[^ ^2^ ^]^ Therefore, the identification and quantification of additional HDL functionality biomarkers are vital to unravel the role of HDL in CVD.

The constant metabolism and remodeling of HDL particles results in circulating HDL particles that differ in functionality and composition irrespective of cholesterol content.^[^ ^2^ ^]^ One of the main functions of HDL appears to be its cholesterol efflux (ChE) capacity. ChE from macrophages to HDL is inversely associated with prevalent coronary artery disease,^[^ ^3^ ^]^ type 2 diabetes mellitus,^[^ ^4^ ^]^ and incident atherosclerotic CVD.^[^ ^5^ ^]^ Therefore, ChE may be a useful biomarker for cardiovascular risk assessment. In addition, HDL resistance to oxidation has been related to its ChE capacity^[^ ^6^ ^]^ and HDL antioxidative activity^[^ ^7^ ^]^ due to the activity of the paraoxonase 1 enzyme.

Lipidomics is an emerging “omic” science that allows the study of several lipid classes and species, in addition to the classical cholesterol and triacylglyceride (TAG) levels determined routinely in clinical laboratories.^[^ ^8^ ^]^ The study of the HDL lipidome may be a valuable approach for identifying new biomarkers of HDL functionality beyond those traditionally used,^[^ ^9^ ^]^ because the lipid content of HDL is estimated to be approximately 35–65% by weight depending on the HDL subclass.^[^ ^10^ ^]^ Its lipid content is mainly located on the amphipathic surface of the HDL particle, forming a monolayer of phospholipids (35–50% of the total HDL lipid mass). This monolayer also contains minor sphingolipids (5–7%), along with free cholesterol and other sterols (5–10%). The hydrophobic core of HDL is mostly composed of cholesteryl esters (CE; 35–40%) and TAG (2–3%).^[^ ^2^, ^10^, ^11^ ^]^

The HDL lipidome is altered in several medical conditions,^[^ ^12^, ^13^ ^]^ such as metabolic syndrome,^[^ ^14^, ^15^ ^]^ coronary artery disease, and type 2 diabetes mellitus.^[^ ^16^ ^]^ Different lipid profiles have also been described in atherosclerotic lesions when compared to those identified in the circulation.^[^ ^8^ ^]^ Therefore, lipidomics is a useful tool for understanding HDL lipid metabolism and possibly for the discovery of novel lipid metabolites associated with CVD, serving as potential new biomarkers for HDL functionality assessment.^[^ ^9^, ^12^ ^]^

The HDL lipidome is also significantly affected by nutritional factors. Dietary fats from different food sources modify the fatty acid (FA) composition of the HDL surface due to the different FA compositions of dietary fats, especially the high content of monounsaturated fatty acids (MUFAs) in olive oil (OO).^[^ ^17^ ^]^ Virgin olive oil (VOO) contains not only MUFAs but also minor components, including phenolic compounds, which represent 1–2% of the total content of VOO.^[^ ^18^ ^]^ Even though phenolic compounds account for a small amount of VOO weight, they are responsible for the cardioprotective benefits of VOO.^[^ ^18^, ^19^, ^20^, ^21^ ^]^ In particular, our group has previously reported that the sustained intake of functional VOOs enriched with phenolic compounds modifies HDL function and composition, specifically the HDL proteome, towards a cardioprotective mode.^[^ ^22^, ^23^ ^]^ However, to the best of our knowledge, no studies have aimed to discern the effects of VOO phenolic compounds on the HDL lipidome.

The main aim of this study is to assess the effects of the sustained intake of VOO and two different functional VOOs, enriched with its own phenolic compounds (mainly secoiridoids) or with its own phenolic compounds plus complementary compounds from thyme (mainly flavonoids) on the HDL lipidome. Moreover, we also investigated whether such changes in the HDL lipidome are associated with changes in HDL functionality, in particular HDL ChE capacity and its resistance to oxidation, as a surrogate of HDL functionality.

## 2 Results

### 2.1 Characteristics of the Study Participants and Dietary Adherence

In the VOHF study, 62 participants were assessed for eligibility and 33 were randomized and therefore allocated into one of the three sequences of intervention (Figure S2, Supporting Information). The baseline characteristics of the participants are described in Table S2, Supporting Information.^[^ ^22^, ^24^ ^]^ As previously published, participant adherence was good and no changes were reported in the main nutrients and medication intake throughout the study.^[^ ^22^, ^25^ ^]^

### 2.2 HDL Lipid and Protein Characterization

The HDL isolated for this study was shown to be pure and to contain no remnants from other lipoproteins, as shown by the ApoB100 and albumin levels in Table S3, Supporting Information. Moreover, some general changes in HDL composition were observed after the three interventions (Table S4, Supporting Information) as previously published by our group.^[^ ^23^ ^]^

### 2.3 Multivariate Analysis of the HDL Lipidome

Samples were randomly analyzed in three different batches run on 3 consecutive days. The multivariate principal component analysis performed in every set of analyses discarded any batch effect (data not shown). Quality controls were included in the analysis every 10 samples. The analysis of such controls showed intra‐assay coefficients of variation <12% and inter‐assay coefficients of variation <15%.

A total of 13 lipid classes, 185 LC‐FA combinations, and 792 lipid species were identified in HDL, as detailed in Table S5, Supporting Information.

Multivariate analyses performed with Metaboanalyst software^[^ ^26^ ^]^ allowed us to identify those lipids that were differentially present in the HDL lipidome before and after each intervention (within‐intervention comparisons) and among the interventions (between‐intervention comparisons). In all the sets of multivariate analyses, the OPLS‐DA showed a trend for separating two different groups of data, corresponding to pre‐ and post‐intervention values in the within‐intervention comparisons, and to the different OO interventions in the between‐intervention comparisons. Moreover, of all the models predicted by OPLS‐DA, 12 models improved data classification as indicated by an R^2^/Q^2^ > 0.7. These models correspond to changes in lipid class concentration and composition (after FVOO intake), lipid class composition (after FVOO intake, and comparing VOO vs FVOOT intake), LC‐FA concentration (after FVOO intake and after FVOOT intake), LC‐FA composition (after VOO intake and after FVOOT intake), lipid species concentration (after VOO intake and comparing VOO vs FVOOT intake), and lipid species composition (after VOO intake, after FVOOT intake, and comparing VOO vs FVOOT intake).

Paired t‐test analyses performed in these 12 data sets with R^2^/Q^2^>0.7 showed that significant changes were found in three of these data sets, as indicated by a *p*‐value adjusted by FDR < 0.1. The OPLS‐DA plots of these three sets of analyses are shown in Figure S3, Supporting Information. These analyses revealed that no changes in LC‐FA concentration, lipid class concentration, or lipid class composition after any intervention were observed. However, the LC‐FA composition of eight lipids, the concentration of five lipid species, and the composition of 54 lipid species were significantly modified after VOO intake. Comprehensive information on the fold change, the *p*‐value, and the *p*‐value adjusted by FDR of these lipids is detailed in **Table** **1**.

**Table 1:** HDL lipids significantly modified after VOO intervention versus its baseline

Lipid	Log fold change	*p*‐value	*p*‐value adjusted by FDR
*LC‐FA combination composition*			
TAG(FA18:1)	0.0940763	0.0001126	0.0124762
SM(FA22:1)	0.1038876	0.0001543	0.0124762
CE(FA22:6)	−0.2134798	0.0002163	0.0124762
PC(FA18:1)	0.1147135	0.0013702	0.0538892
TAG(FA18:2)	−0.1862806	0.0015575	0.0538892
SM(FA26:0)	−0.1462294	0.0026301	0.0703188
CE(FA18:1)	0.0616907	0.0029727	0.0703188
TAG(FA20:1)	0.1643749	0.0032517	0.0703188
*Lipid species concentration*			
TAG56:8(FA18:2)	−0.4777177	0.0000352	0.0270411
PC(FA18:1/FA18:1)	0.3392268	0.0001713	0.0658497
TAG51:4(FA18:2)	−0.3454150	0.0004444	0.0867678
TAG51:5(FA18:3)	−0.7895801	0.0004843	0.0867678
TAG54:7(FA22:5)	−0.7164132	0.0005642	0.0867678
*Lipid species composition*			
TAG56:5(FA20:3)	0.2616832	0.0000099	0.0043817
TAG51:4(FA18:2)	−0.3755527	0.0000126	0.0043817
TAG51:4(FA15:0)	−0.3697035	0.0000171	0.0043817
TAG54:2(FA20:1)	0.2707045	0.0000334	0.0064200
TAG52:2(FA16:0)	0.1733400	0.0000996	0.0135271
TAG52:2(FA18:1)	0.1893192	0.0001055	0.0135271
PC(FA18:1/FA18:1)	0.2851526	0.0001326	0.0145688
SM(FA22:1)	0.1038876	0.0001543	0.0148317
TAG54:7(FA22:5)	−0.8269101	0.0002159	0.0166373
CE(FA22:6)	−0.2134798	0.0002163	0.0166373
TAG56:8(FA18:2)	−0.5077355	0.0003964	0.0277134
TAG51:5(FA18:3)	−0.9335297	0.0005247	0.0336250
TAG54:2(FA18:1)	0.1971140	0.0005983	0.0353917
TAG56:4(FA20:2)	0.2516185	0.0006653	0.0365420
TAG54:4(FA20:3)	0.1716807	0.0007294	0.0373921
TAG56:4(FA20:3)	0.1471088	0.0008224	0.0395282
TAG50:4(FA18:2)	−0.3485138	0.0008820	0.0398996
TAG52:4(FA18:2)	−0.3113580	0.0010850	0.0441748
TAG52:4(FA16:0)	−0.3070039	0.0011523	0.0441748
TAG53:3(FA18:2)	−0.1628848	0.0011950	0.0441748
TAG50:3(FA14:1)	0.2673740	0.0012636	0.0441748
TAG52:1(FA18:1)	0.1685961	0.0012638	0.0441748
TAG54:2(FA16:0)	0.1873376	0.0013449	0.0449658
TAG54:3(FA20:2)	0.1731868	0.0014159	0.0453672
TAG54:3(FA18:1)	0.2509162	0.0019271	0.0574393
TAG54:8(FA18:2)	−0.8899613	0.0019420	0.0574393
TAG51:3(FA18:2)	−0.1743374	0.0023050	0.0648026
TAG51:3(FA15:0)	−0.1886565	0.0023595	0.0648026
TAG54:2(FA18:0)	0.1473225	0.0024488	0.0649346
SM(FA26:0)	−0.1462308	0.0026301	0.0674181
TAG52:5(FA16:0)	−0.3024300	0.0028230	0.0692749
TAG54:6(FA16:0)	−0.2767250	0.0029219	0.0692749
CE(FA18:1)	0.0616905	0.0029728	0.0692749
TAG52:6(FA16:1)	−0.3444020	0.0033464	0.0721775
TAG52:5(FA18:3)	−0.2470372	0.0033575	0.0721775
TAG52:5(FA18:2)	−0.3449757	0.0033789	0.0721775
TAG54:7(FA18:2)	−0.5761714	0.0035175	0.0731075
TAG54:7(FA20:4)	−0.2252724	0.0036766	0.0734343
DAG(FA18:2/FA22:6)	−3.7042677	0.0038030	0.0734343
TAG52:6(FA18:2)	−0.4073001	0.0038197	0.0734343
TAG50:5(FA16:0)	−0.7027341	0.0041405	0.0766631
TAG54:6(FA18:2)	−0.4742435	0.0041871	0.0766631
TAG52:6(FA16:0)	−0.5787183	0.0044123	0.0788637
TAG56:7(FA18:2)	−0.2425078	0.0045124	0.0788637
TAG56:8(FA16:0)	−0.4646922	0.0046963	0.0802540
TAG56:8(FA22:6)	−0.5023336	0.0049195	0.0821218
TAG55:1(FA16:0)	−0.4700962	0.0050191	0.0821218
TAG50:4(FA14:0)	−0.3123558	0.0060180	0.0959950
TAG52:3(FA16:0)	−0.0962283	0.0061255	0.0959950
TAG56:7(FA22:5)	−0.2748293	0.0065134	0.0959950
TAG54:5(FA18:0)	−0.2489313	0.0065471	0.0959950
TAG56:3(FA20:1)	0.2700999	0.0066015	0.0959950
TAG56:3(FA18:1)	0.2131561	0.0066160	0.0959950
TAG54:6(FA20:4)	−0.1866872	0.0068664	0.0977823

Data correspond to paired *t* test analyses of those data sets with R^2^/Q^2^ > 0.7 in the models predicted by OPLS‐DA and with *p*‐value adjusted by FDR < 0.1 in the multivariate analysis. Data is ordered according to the *p*‐value adjusted by FDR. CE, cholesteryl esters; DAG, diacylglycerol; FA, fatty acid; FDR, false discovery rate; PC, phosphatidylcholine; TAG, triacylglycerol; SM, sphingomyelin.

The data corresponding to the lipids with an R^2^/Q^2^ > 0.7 in the models predicted by OPLS‐DA and that were differently present (*p*‐value adjusted by FDR < 0.1) were further analyzed by using both general linear models (within‐intervention comparisons) and ANCOVA (between‐intervention comparisons) adjusted by age, sex, baseline values, the sequence of intervention, and the number of HDL particles, as described below.

### 2.4 Changes in LC‐FA Combination Composition

Table S6, Supporting Information, shows the changes (95% CI) in LC‐FA combination composition (%), after 3‐weeks of sustained intake of VOO, FVOO, and FVOOT. In general, all the LC‐FA combinations that were modulated in the present study were significantly modified after VOO, and most of them were also modified after FVOO. However, these changes were not always significant among them (between intervention differences).

With regard to FA saturation, some MUFAs showed an increase after OO consumption. There was a significant increase in CE(FA18:1) after the VOO, FVOO, and FVOOT interventions. Although the significant quadratic trend (*p* < 0.001) indicates that the increase after FVOO tends to be higher than that after FVOOT, no differences were observed among interventions. The same occurred for SM(FA22:1). The MUFA 18:1 presence in PC and TAG((PC(FA18:1) and TAG(FA18:1), respectively) increased after VOO and FVOO intake, but not after FVOOT. The increase observed after VOO reached a borderline significance (*p* < 0.09) versus the changes after FVOOT. TAG(FA20:1) showed only a borderline significant increase after VOO intervention (*p* = 0.091).

In contrast, one saturated FA (SFA) and two PUFAs decreased after all interventions. There was a significant decrease in the PUFA 22:6 presence in CE (CE(FA22:6)) after the three interventions (*p* < 0.05), and the decrease after VOO was significant versus that after FVOO intake (*p* = 0.015). The presence of PUFA 22:6 in SM and PUFA 18:2 in TAG (SM(FA22:6) and TAG(FA18:2), respectively) decreased reaching significance only after the VOO and FVOO interventions (*p* < 0.05). In the former, no differences among interventions were observed. In the latter, however, the decreases observed after VOO and FVOO intake reached significance versus the changes after FVOOT (*p* < 0.05).

### 2.5 Changes in Lipid Species Concentration

Table S7, Supporting Information, shows the changes (95% CI) in lipid species concentrations (nmol g^–1^) after 3‐weeks of sustained intake of VOO, FVOO, and FVOOT. In general, an increase in some lipid species containing MUFAs and a decrease in lipid species containing PUFAs were observed after the interventions.

In particular, PC(FA18:1/FA18:1) increased after the three interventions (*p* < 0.05). Although the significant quadratic trend (*p* < 0.001) indicates that the increase after VOO or FVOO tends to be higher than that after FVOOT, no significant differences were observed among interventions. TAG51:4(FA18:2) decreased significantly only after VOO and FVOO intake (*p* < 0.05), but no differences were observed among interventions. In the case of TAG56:8(FA18:2), a significant decrease was present only after VOO intervention, and this decrease reached significance versus the changes observed after FVOOT intervention (*p* = 0.007). No significant changes in either or between intervention TAG54:7(FA22:5) levels were observed, likely due to the minute abundance of this lipid species.

### 2.6 Changes in Lipid Species Composition

In general, changes were observed in SM, CE, DAG, and PC lipid species, but TAG was the lipid class that showed the most significant changes after OO intervention.

#### 2.6.1 SM, CE, DAG, and PC Lipid Species Composition

Changes in SM and CE lipid species composition have been previously described in the “Changes in LC‐FA combination composition” section and in Table S6, Supporting Information. Table S8, Supporting Information, shows the changes (95% CI) in DAG and PC lipid species composition (%) after 3 weeks of sustained intake of VOO, FVOO, and FVOOT. In general, an increase in those lipid species containing MUFAs and a decrease in those containing PUFAs or SFAs were observed.

Between‐intervention comparisons showed an increase in DAG(FA18:2/FA22:6) after FVOOT versus VOO and FVOO (*p* < 0.05). As observed when the concentration data were examined, the PC(FA18:1/FA18:1) composition increased after all interventions (*p* < 0.05). Although the significant quadratic trend (*p* < 0.001) indicates that the increase after VOO or FVOO tends to be higher than that after FVOOT, no differences were observed among interventions.

#### 2.6.2 TAG Lipid Species Composition

##### Increase After Consumption

Table S9, Supporting Information, shows the increases (mean, 95% CI) in TAG lipid species composition (%) after 3‐weeks of sustained intake of VOO, FVOO, and FVOOT.

In general, almost all TAGs that increased contained MUFAs (*n* = 8) but some TAGs containing PUFAs (*n* = 5) and SFAs (*n* = 3) also increased after OO intake. Moreover, all the TAG lipid species that increased in the present study, increased after VOO and some of them also increased after FVOO but these changes were not always significant among them (between intervention differences). No TAG specific significant increases were observed after FVOOT intake. These changes can be comprehensively explained allocating them into three different patterns.

In the first pattern, some lipid species’ compositions increased significantly after VOO and FVOO intake, but these changes were not statistically significant compared to those observed after FVOOT (between intervention differences). In this group the following lipid species are included: TAG50:3(FA14:1); TAG52:1(FA18:1); TAG54:2(FA18:1); TAG54:2(FA20:1); TAG54:3(FA18:1); TAG56:3(FA20:1); TAG56:4(FA20:3), and TAG56:5(FA20:3).

In the second pattern, similar to the previous pattern, significant increases occurred after VOO and FVOO intake, and these increases were significant versus the changes after FVOOT intervention (*p* < 0.03). In this group, two lipid species are included: TAG52:2(FA16:0) and TAG52:2(FA18:1).

In the third pattern, some lipid species’ compositions increased only after VOO intake, but these changes were not significantly different from those observed after FVOO and FVOOT (between intervention differences). In this pattern the following lipid species are included: TAG54:2(FA16:0); TAG54:2(FA18:0); TAG54:3(FA20:2); TAG4:4 (FA20:3); TAG56:3(FA18:1), and TAG56:4(FA20:2).

###### Decrease after Consumption

Table S10, Supporting Information, shows the decreases (mean, 95% CI) in TAG lipid species composition (%) after 3 weeks of sustained intake of VOO, FVOO, and FVOOT.

In general, almost all TAG that decreased contained PUFAs (*n* = 18) or SFAs (*n* = 11), and only one TAG containing MUFAs decreased after OO intake. Moreover, most of the TAG lipid species that decreased in the present study, decreased after VOO, after FVOO, or after the intake of both OOs. These changes were significant among them in some cases (between intervention differences). Different patterns of effects arise from these results as detailed below.

In the first pattern, some lipid species decreased significantly only after FVOO intake. Between‐intervention analyses showed that the decreases, not only after FVOO intake but also after VOO intake, were significant, or reached a borderline significance, versus the changes after FVOOT intake. In this pattern, the following lipid species are included: TAG50:4(FA14:0); TAG52:6(FA18:2); TAG54:6(FA16:0); TAG54:6(FA18:2), and TAG54:7(FA18:2).

In the second pattern, a decrease after VOO and FVOO occurred, and differences after FVOOT consumption reached significance versus VOO, FVOO, or both (*p* < 0.05). In this group the following lipid species are included: TAG50:4(FA18:2); TAG51:3(FA15:0); TAG51:4(FA15:0); TAG52:4(FA16:0); TAG52:4(FA18:2); TAG52:5(FA16:0); TAG52:5(FA18:2); TAG52:5(FA18:3); TAG52:6(FA16:1); TAG54:7(FA20:4); TAG56:7(FA18:2), and TAG56:8(FA18:2). In this group TAG54:8(FA18:2) could also be included, although intra‐intervention differences after FVOO did not reach significance, probably due to the small sample size.

The third pattern observed in Table S10, Supporting Information, is similar to the second pattern because some lipid species decreased after VOO and FVOO intake, but no differences or borderline ones were observed between FVOOT intake versus the other interventions. In this group, three lipid species are included: TAG51:4(FA18:2); TAG52:3(FA16:0); and TAG54:6(FA20:4).

In the fourth pattern, some lipid species decreased after the three interventions, including TAG51:3(FA18:2) and TAG53:3(FA18:2). For the former, no differences between interventions were observed, whereas for the latter decreases after FVOO were significant versus those observed after FVOOT (*p* = 0.035).

In the fifth pattern, the changes in some lipid species reached significance only in the between‐intervention comparisons as in the case of the following lipid species: TAG54:5(FA18:0); TAG55:1(FA16:0); TAG56:7(FA22:5); and TAG56:8(FA16:0). The significant quadratic trends found in the within‐intervention comparisons, however, match the borderline significances (*p* < 0.1) obtained in the between‐intervention comparisons: in general, a higher decrease after FVOOT intake was observed versus VOO consumption. In the case of TAG54:5(FA18:0), between‐intervention changes were also significant between FVOOT and FVOO intake (*p* = 0.038). TAG52:6(FA16:0) could be included in this pattern, although the between‐intervention differences after FVOOT versus VOO intake did not reach significance, probably due to the small sample size.

### 2.7 Associations of Changes in the HDL Lipidome with Changes in HDL Functionality: HDL ChE Capacity and HDL Resistance to Oxidation

Lipids with an R^2^/Q^2^ > 0.7 in the models predicted by OPLS‐DA and that were differently present (*p*‐value adjusted by FDR < 0.1) were further analyzed to assess their relationship with changes in HDL functionality (ChE and HDL resistance to oxidation), as described below in depth.

The Pearson's correlation coefficients of the 3‐week changes in lipids differently modified after OO intake and the HDL functionality are detailed in Tables S11, Supporting Information, to S15 (ChE) and Tables S16, Supporting Information, to S20 (resistance to oxidation). The superscripts in these tables indicate cases where Pearson's correlations were nonsignificant, but Spearman's correlations were significant. Variables with significant (*p* < 0.05) or borderline (*p* < 0.1) values in the correlation coefficients were entered into a stepwise linear regression model as detailed in the Statistics section and described below.

### 2.8 HDL ChE Capacity

**Table** **2** shows the lipid associations of the 3‐week changes in the HDL lipidome and ChE capacity in hypercholesterolaemic subjects after OO ingestion. As this table shows, different variables were associated with the 3‐week changes in ChE after each type of OO.

**Table 2:** Associations of 3 week changes in ChE and HDL lipidome in hypercholesterolemic subjects after OOs ingestion

Predictor variable (3 week changes)	B coefficient	SE	Standardized B	T	*p*‐value
***VOO (n = 30)***					
TAG51:3(FA15:0), %	−42.7	11.4	−0.546	−3.72	**0.001**
TAG52:2(FA18:1), %	−0.19	0.08	−0.362	−2.31	**0.029**
**F*VOO (n = 31)***					
TAG54:6(FA18:2), %	−0.89	0.47	−0.309	−1.89	***0.070***
***FVOOT (n = 30)***					
TAG56:3(FA20:1), %	14.6	8.41	0.334	1.73	***0.095***

Stepwise linear regression model adjusted by age and sex. For all oils, a stepwise general linear mixed model was fitted adjusted by age and sex and individual level of test subjects as a random effect. Significant values in **bold** and borderline ones in ***italic bold***. SE, standard error; TAG, triacylglycerol.

Concerning VOO changes, the TAG51:3(FA15:0) 3 week changes showed an inverse association with those observed for ChE (*p* = 0.001). Therefore, the decrease observed in TAG51:3(FA15:0) after VOO intake (Table S10, Supporting Information) is associated with an increase in ChE. The TAG52:2(FA18:1) 3 week changes also showed an inverse relationship with those observed for ChE (*p* = 0.029). This implies, however, that the increase observed in TAG52:2(FA18:1) after VOO may be associated with a decrease in ChE.

With regard to FVOO, only the lipid species TAG54:6(FA18:2) was inversely associated in a borderline way (*p* = 0.070) with changes in ChE after FVOO ingestion. Thus, the decrease observed in TAG54:6(FA18:2) after FVOO ingestion (Table S10, Supporting Information) could be associated with an increase in ChE.

Concerning FVOOT, TAG56:3(FA20:1) was directly associated with changes in ChE with a borderline significance (*p* = 0.095). Accordingly, we observed an increase, but without significance, after FVOOT consumption (Table S10, Supporting Information) which could be associated with an increase in ChE.

### 2.9 HDL Resistance to Oxidation

**Table** **3** shows the lipid associations of the 3‐week changes in HDL lipidome and HDL resistance to oxidation in hypercholesterolaemic subjects after OO ingestion. As this table shows, different variables were associated with the 3‐week changes in HDL resistance to oxidation after each type of OO.

**Table 3:** Associations of 3‐week changes in HDL resistance to oxidation and HDL lipidome in hypercholesterolemic subjects after OOs ingestion

Predictor variable (3‐week changes)	B coefficient	SE	Standardized B	T	*p*‐value
***VOO (n = 21)***					
SM(FA22:1), %	11.8	5.50	0.386	2.14	**0.048**
TAG52:3(FA16:0), %	−20.5	7.40	−0.504	−2.77	**0.014**
***FVOO (n = 15)***					
TAG(FA18:2), %	−7.64	1.96	−0.698	−3.89	**0.003**
TAG52:5(FA18:2), %	−150	63	−0.537	−2.40	**0.035**
***FVOOT (n = 22)***					
TAG52:2(FA18:1), %	−3.50	1.44	−0.471	−2.43	**0.025**

Stepwise linear regression model adjusted by age and sex. For all oils, a stepwise general linear mixed model was fitted adjusted by age and sex and individual level of test subjects as a random effect. Significant values in **bold**. SE, standard error; TAG, triacylglycerol.

Concerning VOO changes, the 3 week changes in SM(FA22:1) were directly associated with changes in HDL resistance to oxidation (*p* = 0.048), whereas the changes in TAG52:3(FA16:0) were inversely associated with changes in HDL resistance to oxidation (*p* = 0.014). Thus, the increase in SM(FA22:1) (Table S6, Supporting Information) and the decrease in TAG52:3(FA16:0) (Table S10) observed after VOO consumption are associated with an increase in the HDL resistance to oxidation.

After FVOO consumption, the 3 week changes in the LC‐FA combination TAG(FA18:2) and in the lipid species TAG52:5(FA18:2) both appear to be inversely associated with changes in HDL resistance to oxidation (*p* = 0.003 and *p* = 0.035). Thus, the decrease observed in both of them after FVOO consumption (Tables S6 and S10, Supporting Information) is associated with an increase in the HDL resistance to oxidation.

After FVOOT consumption only the increase in TAG52:2(FA18:1) was inversely associated with changes in HDL resistance to oxidation (*p* = 0.025). However, the increase in this lipid species observed after FVOOT intake did not reach significance (Table S10, Supporting Information).

## 3 Discussion

This study shows that the sustained intake of VOO and two different functional VOOs, enriched with its own phenolic compounds (mainly secoiridoids; FVOO) or with its phenolic compounds plus complementary compounds from thyme (mainly flavonoids; FVOOT) differentially modulates the HDL lipidome. In general, VOO and FVOO similarly increase the presence of MUFAs in several lipid classes and species, at the expense of decreasing SFAs and PUFAs, especially in CE, SM, PC, DAG, and TAG. The intake of FVOOT, did not induce the same lipid changes observed after VOO and FVOO intake, even though they had the same lipid matrix (Table S1, Supporting Information). Interestingly, most of the significant changes associated with HDL were observed in TAG species, despite it being a minor lipid class present in the HDL particle as it accounts for only 2–3% of the total HDL lipid mass.^[^ ^27^ ^]^

According to the results observed after the sustained intake of VOOs, dietary fats from different sources modify the HDL lipid composition.^[^ ^17^ ^]^ A diet rich in MUFAs modifies the chain length of the phospholipids embedded in the HDL which, in turn, promotes changes in HDL monolayer fluidity and eventually enhances HDL ChE capacity when compared to diets rich in SFAs or PUFAs.^[^ ^28^ ^]^ Moreover, OO intake also modifies the FA profile of the PC present on the HDL surface, increasing MUFAs, and decreasing SFAs.^[^ ^17^ ^]^ These changes were assumed to be due to the different FA compositions of the dietary fats consumed, especially the high content of MUFAs in OO.^[^ ^17^, ^29^ ^]^ In the present study, the intake of different VOOs with the same FA profile but with different phenolic compound profiles modified the HDL lipidome. However, significant changes in particular LC‐FA combinations and lipid species present in the HDL particles observed after the intake of VOO and FVOO were not different between these OOs. This fact suggests that a quantitative phenolic compound enrichment of VOO with its own phenolic compounds in FVOO might not promote a differential remodeling of the HDL lipidome. Likewise, our group reported an important matrix effect on the cardioprotective remodeling of the HDL proteome, as the protein expression modifications observed in the HDL particles were common for the three VOO interventions.^[^ ^23^ ^]^

In reference to the FA present in the common matrix of the VOOs used in our study, 8.2% are PUFAs (mainly linoleic acid, 7.4%), 13.75% SFAs (mainly palmitic acid, 11.2%), and 77.7% MUFAs (mainly oleic acid, 76.8%). Thus, oleic acid (18:1 ω9) is by far the most abundant FA in VOO. The FA that increased the most in the different lipid species, especially TAG, in the HDL particles was FA18:1. Although the lipidomic approach used in this study does not allow us to know the position of the double bond, we assume that FA18:1 detected in our study is oleic acid due to its high abundance in the common matrix. Other MUFAs that also increased during the intervention with VOO and FVOO were FA22:1, FA20:1, and FA14:1, to the detriment of some SFAs and PUFAs. Recently, a randomized crossover study assessed the short‐term effects (4 days) of a fast‐food diet and a Mediterranean diet intervention on HDL lipidomic composition.^[^ ^30^ ^]^ Similar to our results, they observed that FAs in different classes, specifically PC, TAG, and CE became shorter and had fewer double bonds after fast‐food diet intervention, which was rich in SFAs. In contrast, FAs became longer and had more double bonds after the Mediterranean diet intervention, which was rich in MUFAs.^[^ ^30^ ^]^

Additionally, a study assessing the effects of diets rich in ω‐3 FAs and/or phenolic compounds on the HDL lipidome in overweight human subjects with high CVD risk observed an increase in long‐chain PUFA‐containing TAG after the high ω‐3 FA diet. Moreover, the authors also observed a phospholipid profile characterized by a significant increase of medium‐chain FAs after the diet exclusively rich in phenolic compounds (approximately 2900 mg of phenolic compounds). However, the authors did not specify the family of phenolic compounds administered to the volunteers. Moreover, an inverse association between phenolic compounds and TAGs containing long‐chain low unsaturated FAs was observed.^[^ ^31^ ^]^

The intervention with FVOOT resulted in differential effects on the HDL lipidome when compared to VOO and FVOO. This could likely be due to the enrichment of FVOOT with phenolic compounds from thyme (mainly flavonoids). Thus, the presence of flavonoids could have interfered with the VOO common matrix by reducing the incorporation of MUFAs into the different lipid classes of HDLs. For instance, phenolic compounds affect postprandial lipemia decreasing TAG absorption via inhibition of pancreatic lipase activity or decreasing bile acid reabsorption.^[^ ^32^, ^33^, ^34^ ^]^ Moreover, phenolic compounds can decrease the activity of lipolytic enzymes by affecting the emulsification process and thus decreasing fats absorption and inhibiting the synthesis of lipids in the liver, including TAG and FA.^[^ ^33^ ^]^

In summary, the specific type of phenolic compounds (mainly secoiridoids and hydroxytyrosol derivates) and the given dose (25 mL day^‐1^ for 3 weeks) could exert a key role in lipid metabolism,^[^ ^33^, ^34^ ^]^ including HDL, leading to changes in the HDL lipidome. Thus, we hypothesize that thyme flavonoids present in FVOOT at administered dosages could influence fat metabolism, causing a decrease in MUFA incorporation into HDL lipid species when compared with OOs enriched with their own phenolic compounds.

In the present study, we also aimed to elucidate whether the changes in the lipid classes and LC‐FA combination present in the HDL that occurred after VOO, FVOO, and FVOOT intake were associated with changes in HDL functionality. The vast majority of studies aimed at studying the HDL lipidome are limited to lipid class analysis,^[^ ^14^, ^35^, ^36^, ^37^, ^38^ ^]^ while only a few have gone further relating lipid species and LC‐FA combinations to HDL function.^[^ ^16^, ^39^ ^]^ Our results indicate that changes in specific LC‐FA combinations and lipid species are determinants of HDL ChE capacity and its resistance to oxidation, as a surrogate of HDL functionality. On the one hand, the changes observed after the intake of VOO and FVOO could promote a beneficial increase in the HDL resistance to oxidation in hypercholesterolemic patients, in particular the changes in SM(FA22:1), TAG(FA18:2), TAG52:3(FA16:0) and TAG52:5(FA18:2). On the other hand, some of the changes observed after the intake of VOO and FVOO could enhance HDL ChE capacity in the same population, in particular TAG51:3(FA15:0) and TAG54:6(FA18:2). Once more, these effects observed after VOO and FVOO intake differ from those obtained after FVOOT intake, because some lipids were found to be determinants of ChE and HDL resistance to oxidation after FVOOT intake, but no changes in these lipids were observed after this OO intake.

Ståhlman et al. revealed that dyslipidemia is the major factor affecting functional components in the HDL lipidome in type 2 diabetes mellitus. In particular, an increase in TAG52:3, TAG52:5, and TAG54:6, among others was reported in dyslipidemic type 2 diabetes mellitus subjects when compared with healthy and with normolipidemic type 2 diabetes mellitus subjects.^[^ ^40^ ^]^ In our study carried out in hypercholesterolemic subjects, the TAG52:3(FA16:0) and TAG52:5(FA18:2) decreased after VOO and FVOO intake, and this decrease inversely correlated with HDL resistance to oxidation. This relationship between changes in these TAG species and HDL function was confirmed by the fact that TAG52:3(FA16:0) and TAG52:5(FA18:2) were determinants of HDL resistance to oxidation. Moreover, the lipid species TAG54:6(FA18:2) decreased after FVOO intake, and this decrease inversely correlated with HDL ChE capacity and HDL resistance to oxidation. The relation between the changes in this TAG species and HDL ChE capacity was confirmed by the fact that TAG54:6(FA18:2) was a determinant of HDL function. The decreases in these three lipid species (TAG52:3(FA16:0) after VOO, and TAG52:5(FA18:2) and TAG54:6(FA18:2) after FVOO) observed in our study were associated with an increase in HDL functionality. These species are increased in dyslipidemic type 2 diabetes mellitus subjects.^[^ ^40^ ^]^ Thus, our findings point to an atherogenic role for these abovementioned lipid species. Consequently, the HDL targeted lipidomic assessment of these 3 FA lipid species, which account for only a 6.90% of the TAG fraction of the HDL lipidome, could be a good approach to assess HDL function, in particular ChE and resistance to oxidation as a surrogate of HDL functionality. However, more studies are warranted on this issue.

We have previously reported that changes in lipids present in HDL (other than phospholipids), namely, free cholesterol and TAG, are determinants of HDL fluidity and oxidative status, in turn, determine HDL ChE capacity.^[^ ^6^ ^]^ In concordance with such results, in the present study, we have reported that changes in the SM and TAG composition are prone to modulate both HDL ChE capacity and HDL oxidative status. Concordantly, we have previously reported the enrichment of lipo‐ and hydrophilic antioxidants linked to HDL, particularly phenolic compounds (thymol sulfate, caffeic acid sulfate, hydroxyphenylpropionic acid sulfate, and hydroxytyrosol acetate sulfate) and fat‐soluble antioxidants (alpha‐tocopherol, lutein, ubiquinol and β‐cryptoxanthin), after the intake of FVOO and FVOOT.^[^ ^41^ ^]^ The coexistence of these lipophilic and hydrophilic antioxidants linked to HDL may confer benefits by protecting HDL itself from oxidative damage via different antioxidant pathways.^[^ ^22^ ^]^ The proper oxidative balance in the HDL lipidome may result in an increase in its ChE capacity.

A limitation of this study could be the inability to evaluate whether possible interactions of other dietary components or medication with the intervention OOs modify the HDL lipidome, however controlling diet and medication during the study without any observed changes throughout the intervention could limit these interactions.

In conclusion, lipidomic analyses show that the intake of VOO, FVOO, and FVOOT, differentially modulates the presence of particular FA and lipid species, mostly TAG, in the HDL particle. The changes observed after VOO intake and after FVOO intake were similar, whereas FVOOT did not induce the same FAs changes. The type of phenolic compound present in the OOs, rather than the common matrix of the three OOs, could account for this fact. Moreover, in the present study, we demonstrate that changes in the HDL lipidome in hypercholesterolemic patients are associated with a modulation of HDL functionality promoting an increase in ChE capacity and in HDL resistance to oxidation through a decrease in TAG52:3(FA16:0), TAG52:5(FA18:2), and TAG54:6(FA18:2) lipid species.

In summary, from our data, the type of phenolic compounds present in the same matrix, in our case VOO, modulates the HDL lipidome. Our data also indicate that the HDL lipidome and function are interconnected. Therefore, the assessment of the HDL lipidome could be a good approach to identify and characterize new HDL functionality biomarkers.^[^ ^9^ ^]^ Therefore, therapeutic strategies aimed at reducing CVD risk may take into account not only changes in HDL‐C levels and HDL functionality but also changes in the HDL lipidome.

## 4 Experimental Section

### Phenol‐Enriched OO Preparation and Composition

A natural VOO containing 80 mg kg^−1^ of phenolic compounds was used as a control condition. As described previously, this VOO was also used as a matrix to prepare a functional VOO (FVOO) enriched with its own phenolic compounds, mainly secoiridoid derivatives (500 mg kg^−1^). The same parental VOO was used to prepare a second functional OO (FVOOT; 500 mg kg^−1^) enriched with both its own phenolic compounds (50%; mainly secoiridoid derivatives) and complementary compounds from thyme (50% mainly flavonoids, phenolic acids, and monoterpenes).^[^ ^42^ ^]^ As previously reported, the OOs did not differ in fat and micronutrient composition, except for the phenolic content (Table S1, Supporting Information).^[^ ^25^ ^]^

### Study Subjects, Design of the Study, and Dietary Adherence

The virgin olive oil and HDL functionality (VOHF) study is a randomized, controlled, double‐blind, crossover trial that was conducted in 33 hypercholesterolaemic subjects (total cholesterol >200 mg dL^−1^). Participants ingested 25 mL day^–1^ of raw VOO (control VOO, FVOO, or FVOOT) during meals for 3 weeks, according to the assigned sequence of intervention. All interventions were preceded by 2 week washout periods with common OO (Figure S1, Supporting Information).

The institutional ethics committee (CEIC‐IMAS 2009/3347/I) approved the protocol. The present clinical trial was conducted in accordance with the Helsinki Declaration and the Good Clinical Practice for Trials on Medical Products in the European Community and International Conference of Harmonization. The subjects gave their written informed consent before participation. The study was registered at the International Standard Randomized Controlled Trial Register (Identifier: ISRCTN77500181).

Detailed information on the study subjects, the design of the study, and dietary adherence is described in the Supporting Methods section.

### HDL Isolation and Characterization

Blood was collected in Vacutainer tubes with K2EDTA anticoagulant. Blood samples were centrifuged at 1500 × *g* for 15 min and 2.8 mL of plasma were finally recovered. A protease inhibitor cocktail (Sigma‐Aldrich, Tres Cantos, Spain) was added to plasma at a concentration 1/100 (1 µL of protease inhibitor cocktail for 100 µL of plasma). All samples were stored at −80 °C until HDL isolation. The HDL fraction (1.036–1.21 g mL^−1^) was isolated from plasma by the sequential density gradient ultracentrifugation method as previously described.^[^ ^23^ ^]^ To ensure the purity of the HDL, apolipoprotein(Apo) B100 and albumin levels were determined in these samples by immunoturbidimetric methods using a Cobas‐Mira Plus automated analyzer.

Moreover, HDL lipid and protein characterizations were also performed by enzymatic and immunoturbidimetric methods (ABX‐HoribaDiagnostics, France; Roche Diagnostic System, Spain; Spinreact, Spain) using the automatic analyzer Cobas‐Mira Plus, as previously described.^[^ ^23^ ^]^

Detailed information on the HDL isolation and characterization is described in the Supporting Methods section.

### Lipidomic Data Analyses

Quantitative lipidomic analysis was carried out using the Lipidyzer platform according to the manufacturers’ instructions. Lipid extraction was carried out by applying the methyl tert‐butyl ether extraction method as described elsewhere.^[^ ^43^ ^]^ Lipid analysis and quantification were carried out as described in detail by Cao et al. and Contrepois et al.^[^ ^44^, ^45^ ^]^ The identified and quantified lipid classes, lipid species, and FAs were reported as .xls sheets. In all the quantifications performed, both the concentration (expressed in nmol g^–1^) and composition (expressed as the percentage of the fraction) were analyzed and reported.

Lipid classes refer to: a) four types of phospholipids: lysophosphatidylcholine, lysophosphatidylethanolamine, phosphatidylcholine (PC), and phosphatidylethanolamine; b) five types of sphingolipids: ceramides, dihydroceramides, hexosylceramides, lactosylceramides, and sphingomyelin (SM); and c) four neutral lipids: cholesteryl esters, free FAs, diacylglycerides (DAGs), and TAGs.

Within every particular lipid class, the lipid species refers to different lipid subclasses that differ in any of the FAs that it contains. As an example, this is the case for the two lipid species DAG(12:0/18:1) and DAG(12:0/18:2).

The Lipidyzer platform also allows us to quantify each FA in each particular lipid class. That is, it quantifies the sum of all lipid species containing a determined FA within a particular class of lipids, regardless of the other FAs it is combined with, if any. From now on, the term Lipid Class‐FA (LC‐FA) combination will be used throughout the manuscript to refer to this quantification. As an example, this is the case for the LC‐FA combination DAG(FA12:0) which encompasses both lipid species DAG(FA12:0/FA18:1) and DAG(FA12:0/FA18:2).

### HDL Functionality Assessment and Particle Number

HDL functionality was measured as HDL resistance to oxidation (assessed as the lag time of conjugated diene formation) and ChE capacity as previously described.^[^ ^25^, ^41^, ^46^ ^]^ HDL particle number assessment was performed by nuclear magnetic resonance in a Vantera clinical spectrometer produced by LipoScience (Raleigh, NC, USA).^[^ ^25^ ^]^

### Sample Size and Power Analysis

A sample size of 30 individuals allows a power of at least 80% to detect a statistically significant difference between groups of 3 mg dL^–1^ of HDL‐C (according to the main aim of the VOHF study) and a standard deviation of 1.9. A dropout rate of 15% and a Type I error of 0.05 (2‐sided) were assumed.

### Statistical Analyses

Lipidomic data were matched and aggregated by metabolite sorting. Within‐ and between‐intervention comparisons were performed. For each comparison, only volunteers with values in the compared interventions were considered. Data were normalized using the mean centering on log2 transformed metabolites. For each comparison, the following analyses were performed: 1) principal component analysis to visualize the global variance of the data sets, to reveal intrinsic similarities in the spectral profiles, and to identify outliers; 2) orthogonal partial least squares‐siscriminant analysis (OPLS‐DA) with a permutation test (*N* = 1000). Here, metabolomic data are the descriptor matrix (X) and the interventions (each compared vs its baseline, and the differences between them) were used pairwise as the response variable (Y). Orthogonal signal correction filters were used to remove the variation in the descriptor matrix that it is unrelated to the response variable and thus assist in the interpretation of the model and the identification of metabolites associated with the response variable; 3) Paired *t* test for comparisons with two groups and analysis of variance (ANOVA) for comparisons of the three groups. *p*‐values were adjusted by the false discovery rate (FDR). Variables in which the FDR‐adjusted *p*‐value was <0.1 in the paired *t* test were selected to test variables in a more advanced model, such as an ANCOVA model adjusted by age sex, sequence, HDL particle number, and baseline values, for assessing whether inter‐ and intra‐treatment differences could be obtained.

Variables with an R^2^/Q^2^ > 0.7 in the models predicted by OPLS‐DA and with an FDR‐adjusted *p*‐value <0.1 in the paired *t* test were further analyzed. In these variables, we applied: a) a general linear model, for within‐intervention comparisons; b) an ANCOVA model (adjusted by age, sex, sequence of OO administration, number of HDL particles, and baseline values) for between‐intervention comparisons; and c) univariate associations examined by Pearson's and Spearman's correlation coefficients to assess their relationship with changes in HDL functionality (ChE capacity) and HDL resistance to oxidation as a surrogate of HDL functionality. Variables with significant (*p* < 0.05) or borderline (*p* < 0.1) values obtained in one of the two coefficients were entered in a stepwise linear regression model adjusted by age and sex with restricted collinearity (variation inflation factor [VIF] <2.5). Graphics and paired *t* tests were generated in the R statistical environment (Version 3.5.2) with different packages from the Comprehensive R Archive Network (CRAN 2017) and Metaboanalyst R.^[^ ^47^ ^]^

## Conflict of Interest

The authors declare no conflict of interest

## Supporting information

Supporting Information

## References

[1] P. Barter , M. Caulfield , M. Eriksson , S. Grundy , J. Kastelein , M. Komajda , J. Lopez‐Sendon , L. Mosca , J. Tardif , D. Waters , C. Shear , J. RevkiN , K. Buhr , M. Fisher , A. Tall , B. Brewer , N. Engl. J. Med. 2007, 357, 2109. 17984165 10.1056/NEJMoa0706628

[2] S. K. Karathanasis , L. A. Freeman , S. M. Gordon , A. T. Remaley , Clin. Chem. 2017, 63, 196. 27879324 10.1373/clinchem.2016.257725

[3] A. Rohatgi , A. Khera , J. D. Berry , E. G. Givens , C. R. Ayers , K. E. Wedin , I. J. Neeland , I. S. Yuhanna , D. R. Rader , J. A. de Lemos , P. W. Shaul , D. Ph , I. J. Neeland , I. S. Yuhanna , D. Ph , D. R. Rader , J. A. De Lemos , P. W. Shaul , A. Rohatgi , A. Khera , J. D. Berry , E. G. Givens , C. R. Ayers , K. E. Wedin , I. J. Neeland , I. S. Yuhanna , D. R. Rader , J. a. de Lemos , P. W. Shaul , N. Engl. J. Med. 2014, 371, 2383. 25404125 10.1056/NEJMoa1409065 PMC4308988

[4] D. Saleheen , R. Scott , S. Javad , W. Zhao , A. Rodrigues , A. Picataggi , D. Lukmanova , M. L. Mucksavage , R. Luben , J. Billheimer , J. J. P. Kastelein , S. M. Boekholdt , K. T. Khaw , N. Wareham , D. J. Rader , Lancet Diabetes Endocrinol. 2015, 3, 507. 26025389 10.1016/S2213-8587(15)00126-6 PMC4648056

[5] P. Mody , P. H. Joshi , A. Khera , C. R. Ayers , A. Rohatgi , J. Am. Coll. Cardiol. 2016, 67, 2480. 27230043 10.1016/j.jacc.2016.03.538 PMC4884307

[6] S. Fernández‐Castillejo , L. Rubió , Á. Hernáez , Ú. Catalán , A. Pedret , R.‐M. Valls , J. I. Mosele , M.‐I. Covas , A. T. Remaley , O. Castañer , M.‐J. Motilva , R. Solá , Mol. Nutr. Food Res. 2017, 1700445. 10.1002/mnfr.201700445 PMC5881001 28887843

[7] B. J. Ansell , K. E. Watson , A. M. Fogelman , M. Navab , G. C. Fonarow , J. Am. Coll. Cardiol. 2005, 46, 1792. 16286161 10.1016/j.jacc.2005.06.080

[8] I. E. Hoefer , S. Steffens , M. Ala‐Korpela , M. Bäck , L. Badimon , M.‐L. Bochaton‐Piallat , C. M. Boulanger , G. Caligiuri , S. Dimmeler , J. Egido , P. C. Evans , T. Guzik , B. R. Kwak , U. Landmesser , M. Mayr , C. Monaco , G. Pasterkamp , J. Tuñón , C. Weber , Eur. Heart J. 2015, 36, 2635. 26049157 10.1093/eurheartj/ehv236

[9] F. M. Sacks , M. K. Jensen , Arterioscler. Thromb. Vasc. Biol. 2018, 38, 487. 29371248 10.1161/ATVBAHA.117.307025 PMC5823773

[10] A. Kontush , M. Lindahl , M. Lhomme , L. Calabresi , M. J. Chapman , W. S. Davidson , Contents, High Density Lipoproteins: From Biological Understanding to Clinical Exploitation, Springer International Publishing, London 2015.

[11] H. Y. Choi , A. Hafiane , A. Schwertani , J. Genest , Can. J. Cardiol. 2016, 33, 1.28024548

[12] T. A. Lydic , Y.‐H. Goo , Clin. Transl. Med. 2018, 7, 4. 29374337 10.1186/s40169-018-0182-9 PMC5786598

[13] J. Salazar , L. C. Olivar , E. Ramos , M. Chávez‐Castillo , J. Rojas , V. Bermúdez , Cholesterol 2015, 2015, 1. 10.1155/2015/296417. PMC4655037 26634153

[14] A. A. Khan , P. A. Mundra , N. E. Straznicky , P. J. Nestel , G. Wong , R. Tan , K. Huynh , T. W. Ng , N. A. Mellett , J. M. Weir , C. K. Barlow , Z. H. Alshehry , G. W. Lambert , B. A. Kingwell , P. J. Meikle , Arterioscler. Thromb. Vasc. Biol. 2018, 38, 438. 29284607 10.1161/ATVBAHA.117.310212

[15] M. Jové , A. Naudí , M. Portero‐Otin , R. Cabré , S. Rovira‐Llopis , C. Bañuls , M. Rocha , A. Hernández‐Mijares , V. M. Victor , R. Pamplona , FASEB J. 2014, 28, 5163. 25169057 10.1096/fj.14-253187

[16] W. Pruzanski , E. Stefanski , F. C. de Beer , M. C. de Beer , A. Ravandi , A. Kuksis , J. Lipid Res. 2000, 41, 1035. 10884283

[17] R. Solà , M. F. Baudet , C. Motta , M. Maillé , C. Boisnier , B. Jacotot , Biochim. Biophys. Acta, Lipids Lipid Metab. 1990, 1043, 43. 10.1016/0005-2760(90)90108-a 2310759

[18] M. I. Covas , R. De la Torre , A. Kafatos , R. M. Lamuela‐Raventos , J. Osada , Nutr. Rev. 2006, 64, 20.

[19] M. I. Covas , Pharmacol. Res. 2007, 55, 175. 17321749 10.1016/j.phrs.2007.01.010

[20] J. M. Lou‐Bonafonte , C. Gabás‐Rivera , M. A. Navarro , J. Osada , Nutrients 2015, 7, 4068. 26024295 10.3390/nu7064068 PMC4488773

[21] M. Fitó , R. de la Torre , M. Farré‐Albaladejo , O. Khymenetz , J. Marrugat , M. Covas , Eur. J. Clin. Nutr. 2004, 58, 955.15164117

[22] A. Pedret , S. Fernández‐Castillejo , R.‐M. Valls , Ú. Catalán , L. Rubió , M. Romeu , A. Macià , M. C. López de Las Hazas , M. Farràs , M. Giralt , J. I. Mosele , S. Martín‐Peláez , A. T. Remaley , M.‐I. Covas , M. Fitó , M.‐J. Motilva , R. Solà , Mol. Nutr. Food Res. 2018, 1800456.

[23] A. Pedret , Ú. Catalán , S. Fernández‐Castillejo , M. Farràs , R.‐M. Valls , L. Rubió , N. Canela , G. Aragonés , M. Romeu , O. Castañer , R. de la Torre , M.‐I. Covas , M. Fitó , M.‐J. Motilva , R. Solà , PLoS One 2015, 10, e0129160. 26061039 10.1371/journal.pone.0129160 PMC4465699

[24] M. Farràs , O. Castañer , S. Martín‐Peláez , Á. Hernáez , H. Schröder , I. Subirana , D. Muñoz‐Aguayo , S. Gaixas , R. de la Torre , M. Farré , L. Rubió , Ó. Díaz , S. Fernández‐Castillejo , R. Solà , M. J. Motilva , M. Fitó Colomer , Mol. Nutr. Food Res. 2015, 59, 1758. 26011257 10.1002/mnfr.201500030

[25] S. Fernández‐Castillejo , R.‐M. Valls , O. Castañer , L. Rubió , Ú. Catalán , A. Pedret , A. Macià , M. L. Sampson , M.‐I. Covas , M. Fitó , M.‐J. Motilva , A. T. Remaley , R. Solà , Ú. Catalan , A. Pedret , A. Macià , M. L. Sampson , M.‐I. Covas , M. Fitó , M.‐J. Motilva , A. T. Remaley , R. Solà , Mol. Nutr. Food Res. 2016, 60, 1544. 26992050 10.1002/mnfr.201501068 PMC4949304

[26] Z. Pang , J. Chong , S. Li , J. Xia , Metabolites 2020, 10. 10.3390/metabo10050186. PMC7281575 32392884

[27] A. Kontush , M. Lhomme , J. Glycomics Lipidomics 2015, 05. 10.4172/2153-0637.1000133.

[28] R. Sola , C. Motta , M. Maille , M. T. Bargallo , C. Boisnier , J. L. Richard , B. Jacotot , Arterioscler. Thromb. 1993, 13, 958. 8318513 10.1161/01.atv.13.7.958

[29] R. Solà , C. Motta , M. Maille , M. Bargalló , J. Richard , B. Jacotot , Atheroscler. Thromobosisbosis 1993, 13, 958. 10.1161/01.atv.13.7.958 8318513

[30] C. Zhu , L. Sawrey‐Kubicek , E. Beals , R. L. Hughes , C. H. Rhodes , R. Sacchi , A. M. Zivkovic , Metabolomics 2019, 15, 114. 10.1007/s11306-019-1579-1. 31422486

[31] I. Bondia‐Pons , P. Pöhö , L. Bozzetto , C. Vetrani , L. Patti , A.‐M. Aura , G. Annuzzi , T. Hyötyläinen , A. A. Rivellese , M. Orešič , Mol. Nutr. Food Res. 2014, 58, 1873. 10.1002/mnfr.201400155. 24961394

[32] C. Desmarchelier , P. Borel , D. Lairon , M. Maraninchi , R. Valéro , Nutrients 2019, 11. 10.3390/nu11061299. PMC6627366 31181761

[33] N. Kardum , M. Glibetic , in Adv. Food Nutr. Res., Vol. 84, Academic Press Inc. 2018, 103. 29555067 10.1016/bs.afnr.2017.12.001

[34] B. Pokimica , M. T. García‐Conesa , M. Zec , J. Debeljak‐Martačić , S. Ranković , N. Vidović , G. Petrović‐Oggiano , A. Konić‐Ristić , M. Glibetić , Nutrients 2019, 11, 850. 10.3390/nu11040850. PMC6520894 30991718

[35] J. Salazar , L. C. Olivar , E. Ramos , M. Chávez‐Castillo , J. Rojas , V. Bermúdez , Cholesterol 2015, 2015, 296417. 26634153 10.1155/2015/296417 PMC4655037

[36] M. Holzer , R. Birner‐Gruenberger , T. Stojakovic , D. El‐Gamal , V. Binder , C. Wadsack , A. Heinemann , G. Marsche , J. Am. Soc. Nephrol. 2011, 22, 1631. 21804091 10.1681/ASN.2010111144 PMC3171935

[37] F. Rached , R. D. Santos , L. Camont , M. H. Miname , M. Lhomme , C. Dauteuille , S. Lecocq , C. V. Serrano , M. J. Chapman , A. Kontush , J. Lipid Res. 2015, 55, 2509. 10.1194/jlr.M051631 PMC4242444 25341944

[38] F. Brites , M. Martin , I. Guillas , A. Kontush , BBA Clin. 2017, 8, 66. 28936395 10.1016/j.bbacli.2017.07.002 PMC5597817

[39] E. Zakiev , F. Rached , M. Lhomme , M. Darabi‐Amin , M. Ponnaiah , P. H. Becker , P. Therond , C. V. Serrano , R. D. Santos , M. J. Chapman , A. Orekhov , A. Kontush , J. Clin. Lipidol. 2019, 13, 468. 10.1016/j.jacl.2019.02.004. 31003938

[40] M. Ståhlman , B. Fagerberg , M. Adiels , K. Ekroos , J. M. Chapman , A. Kontush , J. Borén , Biochim. Biophys. Acta, Mol. Cell Biol. Lipids 2013, 1831, 1609. 10.1016/j.bbalip.2013.07.009 23896361

[41] M. Farràs , S. Fernández‐Castillejo , L. Rubió , S. Arranz , Ú. Catalán , I. Subirana , M.‐P. Romero , O. Castañer , A. Pedret , G. Blanchart , D. Muñoz‐Aguayo , H. Schröder , M.‐I. Cova , R. de la Torre , M.‐J. Motilva , R. Solà , M. Fitó , M.‐I. Covas , R. de la Torre , M.‐J. Motilva , R. Solà , M. Fitó , J. Nutr. Biochem. 2018, 51, 99. 10.1016/j.jnutbio.2017.09.010. 29125992

[42] L. Rubió , M. J. Motilva , A. MacIà , T. Ramo , M. P. Romero , J. Agric. Food Chem. 2012, 60, 3105. 22380740 10.1021/jf204902w

[43] A. Körner , E. Zhou , C. Müller , Y. Mohammed , S. Herceg , F. Bracher , P. C. N. Rensen , Y. Wang , V. Mirakaj , M. Giera , Proc. Natl. Acad. Sci. USA 2019, 116, 20623. 31548397 10.1073/pnas.1911992116 PMC6789642

[44] Z. Cao , T. C. Schmitt , V. Varma , D. Sloper , R. D. Beger , J. Sun , J. Proteome Res. 2019. 10.1021/acs.jproteome.9b00289. 31310547

[45] K. Contrepois , S. Mahmoudi , B. K. Ubhi , K. Papsdorf , D. Hornburg , A. Brunet , M. Snyder , Sci. Rep. 2018, 8, 1. 30532037 10.1038/s41598-018-35807-4 PMC6288111

[46] S. Fernández‐Castillejo , L. Rubió , Á. Hernáez , Ú. Catalán , A. Pedret , R.‐M. R.‐M. Valls , J. I. J. I. J. I. Mosele , M.‐I. M.‐I. M.‐I. Covas , A. T. A. T. A. T. Remaley , O. Castañer , M.‐J. M.‐J. Motilva , R. Solá , A. Hernáez , U. Catalán , A. Pedret , R.‐M. R.‐M. Valls , J. I. J. I. J. I. Mosele , M.‐I. M.‐I. M.‐I. Covas , A. T. A. T. A. T. Remaley , O. Castañer , M.‐J. M.‐J. Motilva , R. Solá , Á. Hernáez , Ú. Catalán , A. Pedret , R.‐M. R.‐M. Valls , J. I. J. I. J. I. Mosele , M.‐I. M.‐I. M.‐I. Covas , A. T. A. T. A. T. Remaley , O. Castañer , M.‐J. M.‐J. Motilva , R. Solá , Mol. Nutr. Food Res. 2017, 61. 10.1002/mnfr.201700445. PMC5881001 28887843

[47] J. Chong , M. Yamamoto , J. Xia , Metabolites 2019, 9. 10.3390/metabo9030057. PMC6468840 30909447

