# Supplementary material for: Virgin Olive Oil Phenolic Compounds Modulate the HDL Lipidome in Hypercholesterolaemic Subjects: A Lipidomic Analysis of the VOHF Study
Source: Mol Nutr Food Res. 2021 Mar 18;65(9):2001192. doi: 10.1002/mnfr.202001192 (PMC9286430; doi:10.1002/mnfr.202001192)
Supplement: Supplementary file 1 — Supporting Information [file MNFR-65-0-s001.docx]

**SUPPORTING INFORMATION**

***SUPPORTING METHODS***

***Study subjects***

Hypercholesterolemic (total cholesterol >200 mg/dL) subjects were recruited from newspaper and university advertisements. Volunteers were preselected when their clinical record, physical examination, and blood pressure were within a predefined normal range. Candidates were included in the study when routine hematological and biochemical parameters were within the reference range. Exclusion criteria were the following: low-density lipoprotein (LDL)-cholesterol ≥190 mg/dL, triglycerides ≥350 mg/dL, fasting blood glucose >126 mg/dL, plasma creatinine levels >1.4 mg/dL (women) and >1.5 mg/dL (men), body mass index (BMI)>35, smokers (>1 cigarette/day), athletes with physical activity (>3000 METS.min/day), hypertension, multiple allergies, intestinal diseases, chronic diseases (i.e. diabetes, cardiovascular, etc.), or other conditions that would impair the adherence to the study.

***Design of the study***

The Virgin Olive Oil and HDL Functionality (VOHF) study is a randomized, controlled, double-blind, and crossover trial that was conducted in 33 hypercholesterolemic subjects^[1]^. Participants were instructed to ingest a dose of 25 mL/day of raw VOO (control VOO, FVOO, or FVOOT) during meals for three weeks, according to the assigned sequence of intervention. All interventions were preceded by 2-week washout periods with a common olive oil (**Figure S1**). The selection of the 2-week washout periods was made according to turnover studies, which showed that the mean plasma residence of high-density lipoprotein (HDL) is 4-6 days and that this turnover is not affected by diet^[2]^. Moreover, a 2-week washout period allows the plasma lipid profile to reach equilibrium, as longer intervention periods with fat-rich diets did not modify the lipid concentrations^[3]^. To avoid excessive intake of antioxidants, such as phenolic compounds, during the clinical trial period, participants were advised to limit the consumption of phenol-rich food. Blood samples were collected at the beginning and end of each intervention and sera and plasma EDTA samples were stored at –80ºC until their use. Glucose and lipid profile were measured in plasma EDTA, in a Cobas-Mira Plus (Roche Diagnostic System, Spain). ApoA1, ApoA2, and ApoB100 were measured in plasma EDTA in a PENTRA-400 (ABX-Horiba Diagnostics, France) automated analyzers.

The present clinical trial was conducted in accordance with the Helsinki Declaration and the Good Clinical Practice for Trials on Medical Products in the European Community and International Conference of Harmonization. The institutional ethics committee (CEIC-IMAS 2009/3347/I) approved the protocol. The subjects gave their written informed consent before their participation. The study was registered at the International Standard Randomized Controlled Trial Register (Identifier: ISRCTN77500181).

***Dietary adherence***

We measured 24-h urinary hydroxytyrosol-sulfate and thymol-sulfate, before and after each intervention period as biomarkers of adherence to the FVOO and FVOOT interventions, respectively. Measurements were performed by HPLC- ESI-MS/MS^[1]^. A 3-day dietary record was administered by the participants at baseline and before and after each intervention period to control their habitual diet throughout the study. Participants were asked to avoid a high intake of foods rich in antioxidants (i.e. vegetables, legumes, fruits, etc.). A nutritionist personally advised participants to replace all types of habitually consumed raw fats with the olive oils catered and to limit their rich-polyphenol food consumption.

***HDL isolation and characterization***

The HDL fraction (d=1.036–1.21 g/mL) from the volunteers of the VOHF study was isolated from 2.5 mL of plasma by sequential density gradient ultracentrifugation method as previously described^[4]^. This method employs sodium bromide (NaBr; Sigma-Aldrich, Spain) to isolate different lipoprotein fractions in two consecutive steps. In the firsts step, a fraction containing very low-density lipoprotein (VLDL), intermediate-density lipoprotein (IDL), and LDL, is discarded from the sample by ultracentrifugation (140 000 g for 21 h at 10°C) after adjusting the plasma density to 1.063 g/mL with NaBr. In the second step, the HDL fraction is obtained by ultracentrifugation (140 000 x g for 40 h at 10°C) after the adjustment of the infranatant density to 1.21 g/mL with NaBr. At the end of the process, 2 mL of the HDL fraction is obtained. These samples were stored at -80°C until its use. To assure the purity of the HDL fractions isolated, ApoB100 and albumin levels were determined in these samples by immunoturbidimetric methods in the automated analyzer Cobas-Mira Plus.

Moreover, HDL lipid and protein characterization were also performed by enzymatic and immunoturbidimetric methods (ABX-HoribaDiagnostics, France; Roche Diagnostic System, Spain; Spinreact, Spain) using the automatic analyzer Cobas-Mira Plus, as previously described^[4]^.

***HDL functionality assessment and particle number***

HDL ChE capacity was assessed as previously described in murine J-774A.1 macrophages using a fluorescent cholesterol probe in which the cholesterol molecule is linked to boron dipyrromethene difluoride (BODIPY) moiety (Avanti Polar Lipids, USA)^[5]^. HDL resistance to oxidation was measured as a surrogate of HDL functionality by the conjugated dienes formation after copper oxidation of isolated HDL^[5]^.

HDL particle number was measured by NMR in a Vantera clinical spectrometer, produced by LipoScience (Raleigh, NC, USA) at the National Heart, Lung and Blood Institute, National Institutes of Health (NIH; Bethesda, MD, USA), as previously published^[6]^, to adjust the Analysis of COvariance (ANCOVA) models for between-intervention comparisons.

***SUPPORTING FIGURES***


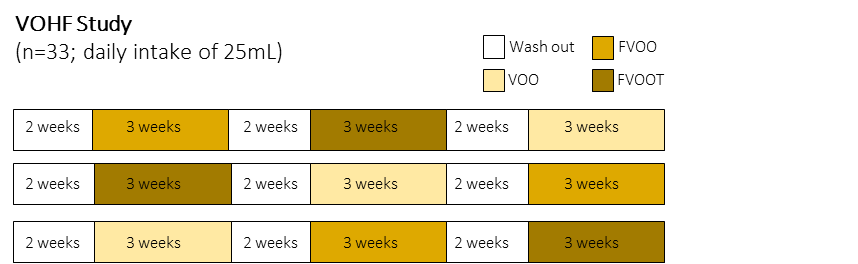


**Figure S1**. **VOHF study design.** Participants were instructed to ingest a daily dose (3 weeks) of 25 mL of control VOO (80 mg/kg), FVOO (500 mg/kg), and FVOOT (250 mg/kg of olive oil phenolic compounds and 250 mg/kg of thyme phenolic compounds). FVOO, functional virgin olive oil enriched with its own phenolic compounds (500 mg/kg); FVOOT, functional virgin olive oil enriched with its own phenolic compounds and complementary ones from thyme at a molar ratio 1:1 (500 mg/kg); VOHF, Virgin Olive Oil and HDL Functionality study; VOO, Virgin Olive Oil.

**
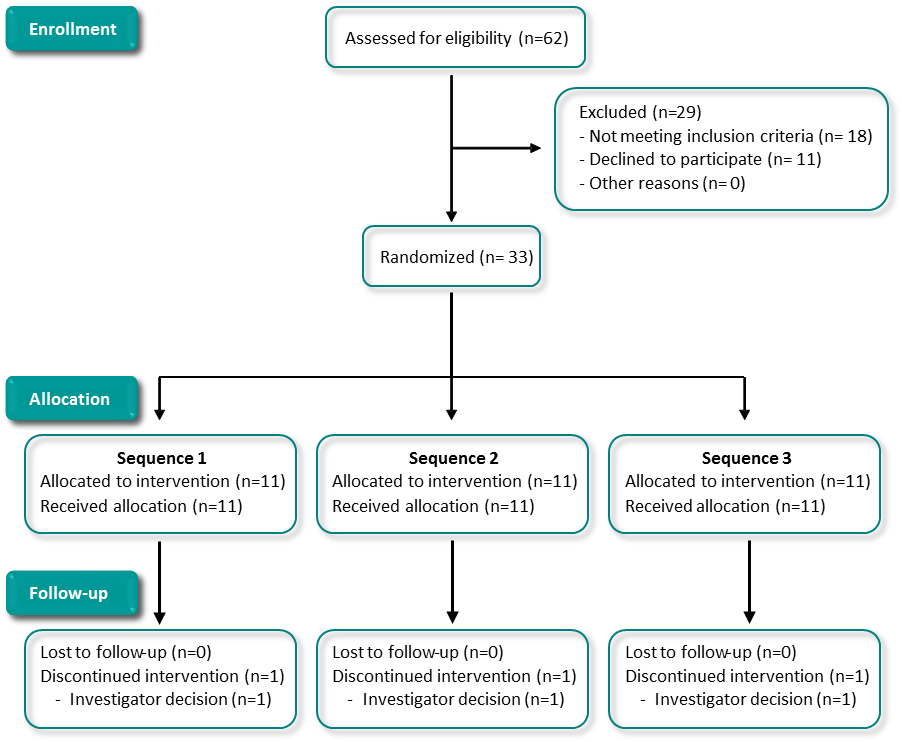
**

**Figure S2. Flowchart of VOHF study.** 62 subjects were assessed for eligibility, and 33 were randomized and therefore allocated in one of the three sequences or intervention.


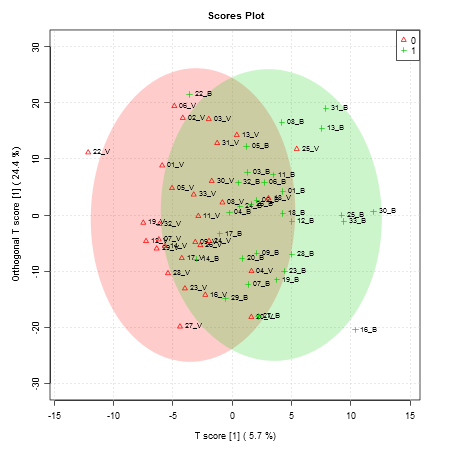


**B**


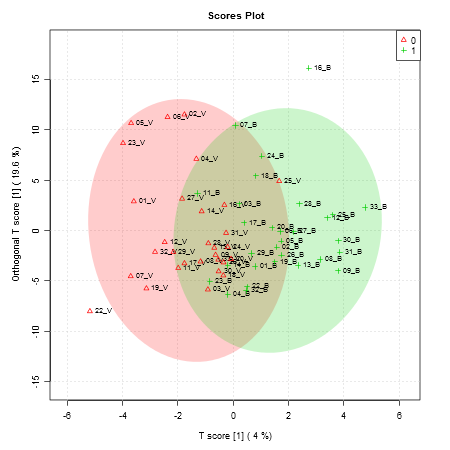


**A**


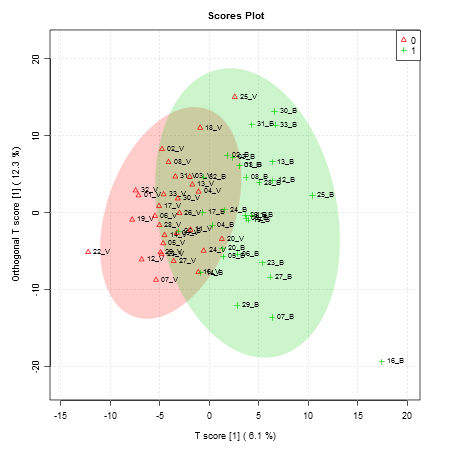


**C**

**Figure S3. OPLD-DA plots of the predicted models with an R^2^/Q^2^ > 0.7 and a p-value adjusted by FDR <0.1.** A) Changes in fatty acid composition after the VOO intervention. B) Changes in lipid species concentration after the VOO intervention. C) Changes in lipid species composition after the VOO intervention. Green and red colors represent basal and post-VOO intervention data, respectively.

***SUPPORTING* TABLES**

**Table S1. Virgin olive oils composition used in the VOHF study. Phenolic compounds, fat soluble micronutrients and fatty acids daily intake through 25 mL of VOO, FVOO and FVOOT.**

|  |  | **VOO** | | | **FVOO** | | | **FVOOT** | | |
| --- | --- | --- | --- | --- | --- | --- | --- | --- | --- | --- |
| **PHENOLIC COMPOUNDS (mg/25 mL/day)** | | | | | | | | | | |
| Hydroxytyrosol | | 0.01 | ± | 0,00 | 0.21 | ± | 0.02 | 0.12 | ± | 0,00 |
| 3,4-DHPEA-AC | | n.d. |  |  | 0.84 | ± | 0.06 | 0.39 | ± | 0.04 |
| 3,4-DHPEA-EDA* | | 0.04 | ± | 0,00 | 6.73 | ± | 0.37 | 3.43 | ± | 0.29 |
| 3,4-DHPEA-EA* | | 0.26 | ± | 0.04 | 0.71 | ± | 0.06 | 0.36 | ± | 0.03 |
| ***Total hydroxytyrosol derivates*** | | ***0.3*** |  |  | ***8.49*** |  |  | ***4.3*** |  |  |
|  | |  |  |  |  |  |  |  |  |  |
| p-hydroxybenzoic acid | | n.d. |  |  | 0.02 | ± | 0,00 | 0.06 | ± | 0,00 |
| Vanillic acid | | n.d. |  |  | 0.07 | ± | 0,00 | 0.13 | ± | 0.01 |
| Caffeic acid | | n.d. |  |  | 0,00 | ± | 0,00 | 0.06 | ± | 0,00 |
| Rosmarinic acid | | n.d. |  |  | n.d. |  |  | 0.41 | ± | 0.03 |
| ***Total phenolic acids*** | | ***-*** |  |  | ***0.09*** |  |  | ***0.65*** |  |  |
|  | |  |  |  |  |  |  |  |  |  |
| Thymol | | n.d. |  |  | n.d. |  |  | 0.64 | ± | 0.05 |
| Carvacrol | | n.d. |  |  | n.d. |  |  | 0.23 | ± | 0.02 |
| ***Total monoterpenes*** | | ***-*** |  |  | ***-*** |  |  | ***0.86*** |  |  |
|  | |  |  |  |  |  |  |  |  |  |
| Luteolin | | 0.04 | ± | 0,00 | 0.18 | ± | 0.02 | 0.21 | ± | 0.02 |
| Apigenin | | 0.02 | ± | 0,00 | 0.06 | ± | 0,00 | 0.1 | ± | 0,00 |
| Naringenin | | n.d. |  |  | n.d. |  |  | 0.2 | ± | 0.02 |
| Eriodictyol | | n.d. |  |  | n.d. |  |  | 0.17 | ± | 0.01 |
| Thymusin | | n.d. |  |  | n.d. |  |  | 1.22 | ± | 0.09 |
| Xanthomicrol | | n.d. |  |  | n.d. |  |  | 0.53 | ± | 0.06 |
| 7-methylsudachitin | | n.d. |  |  | n.d. |  |  | 0.53 | ± | 0.09 |
| ***Total flavonoids*** | | ***0.06*** |  |  | ***0.23*** |  |  | ***2.95*** |  |  |
|  | |  |  |  |  |  |  |  |  |  |
| Pinoresinol | | 0.05 | ± | 0,00 | 0.12 | ± | 0,00 | 0.1 | ± | 0.05 |
| Acetoxipinoresinol** | | 2.47 | ± | 0.19 | 3.66 | ± | 0.31 | 3.24 | ± | 0.28 |
| ***Total lignans*** | | ***2.52*** |  |  | ***3.78*** |  |  | ***3.34*** |  |  |
|  | | | | |  |  |  |  |  |  |
|  | | | | |  |  |  |  |  |  |
| **FAT SOLUBLE MICRONUTRIENTS (mg/25 mL/day)** | | | | | | | | | | |
| α-tocopherol | | 3.27 | ± | 0.01 | 3.4 | ± | 0.02 | 3.44 | ± | 0.01 |
| lutein | | 0.05 | ± | 0,00 | 0.06 | ± | 0,00 | 0.07 | ± | 0,00 |
| β-cryptoxanthin | | 0.02 | ± | 0,00 | 0.03 | ± | 0,00 | 0.02 | ± | 0,00 |
| β-carotene | | 0.01 | ± | 0,00 | 0.02 | ± | 0,00 | 0.02 | ± | 0,00 |
|  | | | | |  |  |  |  |  |  |
|  | | | | |  |  |  |  |  |  |
| **FATTY ACIDS (relative area %)** | | | | | | | | | | |
| Palmitic acid | | 11.21 |  |  | 11.2 |  |  | 11.21 |  |  |
| Stearic acid | | 1.92 |  |  | 1.92 |  |  | 1.92 |  |  |
| Araquidic acid | | 0.36 |  |  | 0.36 |  |  | 0.36 |  |  |
| Behenic acid | | 0.11 |  |  | 0.11 |  |  | 0.11 |  |  |
| ***Total saturated*** | | ***13.75*** |  |  | ***13.74*** |  |  | ***13.75*** |  |  |
|  |  |  |  |  |  |  |  |  |  |  |
| Palmitoleic acid | | 0.7 |  |  | 0.7 |  |  | 0.69 |  |  |
| Oleic acid | | 76.74 |  |  | 76.83 |  |  | 76.75 |  |  |
| Gadoleic acid | | 0.27 |  |  | 0.27 |  |  | 0.27 |  |  |
|  |  |  |  |  |  |  |  |  |  |  |
| ***Total monounsaturated*** | | ***77.71*** |  |  | ***77.8*** |  |  | ***77.72*** |  |  |
| Linoleic acid | | 7.43 |  |  | 7.36 |  |  | 7.43 |  |  |
| Timnodonic acid | | 0.36 |  |  | 0.36 |  |  | 0.35 |  |  |
| Linolenic acid | | 0.43 |  |  | 0.43 |  |  | 0.43 |  |  |
| ***Total polyunsaturated*** | | ***8.22*** |  |  | ***8.15*** |  |  | ***8.22*** |  |  |

Values are expressed as mean ± standard deviation (SD). The acidic composition is expressed as relative area percentage. VOO, virgin olive oil; FVOO, functional VOO enriched with its own phenolic compounds; FVOOT, functional VOO enriched with its own phenolic compounds plus additional complementary ones from thyme; 3,4-DHPEA-AC, 4-(acetoxyethyl)-1,2-dihydroxybenzene; 3,4-DHPEA-EDA, dialdehyde form of elenolic acid linked to hydroxytyrosol; 3,4-DHPEA-EA, oleuropein aglycone; n.d., not detected.

*Quantified with a calibration curve of hydroxytyrosol. **Quantified with a calibration curve of pinoresinol.

**Table S2. Baseline characteristics of the participants of the VOHF study** **(n=33)**

| **Variable** |  | **Sequence 1** | | |  | **Sequence 2** | | |  | **Sequence 3** | | |
| --- | --- | --- | --- | --- | --- | --- | --- | --- | --- | --- | --- | --- |
| Age (y) |  | 54.9 | ± | 12.6 |  | 55.3 | ± | 11.9 |  | 55.5 | ± | 7.8 |
| Gender (male/female)* |  | 5/6 | | |  | 7/4 | | |  | 7/4 | | |
| Hypolipidemic medication: no |  | 7 (63.6%) | | |  | 9 (81.8%) | | |  | 3 (27.3%) | | |
| Body mass index (kg/m^2^) |  | 25.6 | ± | 3.7 |  | 26.3 | ± | 5.2 |  | 27.8 | ± | 4.7 |
| Weight (kg) |  | 74.8 | ± | 16.8 |  | 74.6 | ± | 19.5 |  | 84.5 | ± | 17.7 |
| Systolic blood pressure (mmHg) |  | 125 | ± | 18.7 |  | 128 | ± | 16.7 |  | 130 | ± | 17.9 |
| Diastolic blood pressure (mmHg) |  | 68.1 | ± | 13.5 |  | 72.3 | ± | 9.3 |  | 71.9 | ± | 13.4 |
| Glucose (mg/dL) |  | 88.6 | ± | 11.6 |  | 93.0 | ± | 13.3 |  | 90.9 | ± | 10.5 |
| Total cholesterol (mg/dL) |  | 228.4 | ± | 42.7 |  | 231.9 | ± | 32.7 |  | 218.8 | ± | 31.2 |
| LDL-cholesterol (mg/dL) |  | 150.4 | ± | 32.3 |  | 152.1 | ± | 28.5 |  | 142.3 | ± | 25.7 |
| HDL-cholesterol (mg/dL) |  | 52.8 | ± | 11.8 |  | 53.0 | ± | 12.8 |  | 53.4 | ± | 9.6 |
| Phospholipids (mg/dL) |  | 231.4 | ± | 35.2 |  | 228.9 | ± | 29.1 |  | 222.0 | ± | 16.2 |
| Triglycerides (mg/dL)** |  | 94.00 | | |  | 119.00 | | |  | 117.00 | | |
|  |  | (75.00; 149.00) | | |  | (95.00; 168.00) | | |  | (81.00; 126.00) | | |
| ApoA-I (mg/dL) |  | 142.2 | ± | 22.7 |  | 136.6 | ± | 20.6 |  | 147.5 | ± | 16.3 |
| ApoA-II (mg/dL) |  | 31.9 | ± | 4.7 |  | 30.2 | ± | 3.8 |  | 31.0 | ± | 2.8 |
| ApoB-100 (mg/dL) |  | 115.4 | ± | 20.5 |  | 117.1 | ± | 17.5 |  | 108.7 | ± | 16.2 |

Values are expressed as mean ± SD unless otherwise stated. Sequence 1: FVOO, FVOOT, VOO; Sequence 2: FVOOT, VOO, FVOO; Sequence 3: VOO, FVOO, FVOOT. *p < 0.05 versus Sequence 1.*Values are expressed as number of individuals. **Values are expressed as median (25th; 75th percentiles). Y, years; kg, kilograms; LDL, low-density lipoprotein; HDL, high-density lipoprotein; Apo, apolipoprotein.

**Table S3. ApoB100 and albumin levels in HDL after 3 weeks of sustained intake of VOO, FVOO and FVOOT.**

|  | **VOO** | |  | **FVOO** | |  | **FVOOT** | |  | **Inter-interv p-value** |
| --- | --- | --- | --- | --- | --- | --- | --- | --- | --- | --- |
| **Variable** | **Pre-int** | **Post-int** |  | **Pre-int** | **Post-int** |  | **Pre-int** | **Post-int** |  |  |
|  |  |  |  |  |  |  |  |  |  |  |
| **ApoB100** **(g/L)** | < 0.11 | < 0.11 |  | < 0.11 | < 0.11 |  | < 0.11 | < 0.11 |  | - |
|  |  |  |  |  |  |  |  |  |  | - |
|  |  |  |  |  |  |  |  |  |  | - |
| **Albumin (g/L)** | 1.32 ± 0.85 | 1.22 ± 0.74 |  | 1.11 ± 0.50 | 1.10 ± 0.67 |  | 1.20 ± 0.62 | 1.29 ± 0.78 |  | 0.711 (VOO-FVOOT) |
|  |  |  |  |  |  |  |  |  |  | 0.195 (FVOO-FVOOT) |
|  |  |  |  |  |  |  |  |  |  | 0.404 (VOO-FVOO) |

Values expressed as mean ± S.D. VOO, virgin olive oil; FVOO, functional VOO enriched with its own phenolic compounds; FVOOT, functional VOO enriched with its own phenolic compounds plus additional complementary ones from thyme; HDL, high-density lipoprotein; int, intervention; ApoB100, Apolipoprotein B100. Intra- and inter- intervention p-values were not significant.

**Table S4. HDL composition after 3 weeks of sustained intake of VOO, FVOO and FVOOT.**

|  | **Post-VOO int** | **Post-FVOO int** | **Post-FVOOT int** | **Inter-Int p-value** | **Linear Trend p-value** |
| --- | --- | --- | --- | --- | --- |
|  | **(n=11)** | **(n=11)** | **(n=11)** |  |  |
| **Total cholesterol (mg/dL)** | 31.03 ± 11.06 | 31.77 ± 10.96 | 31.14 ± 11.33 | 0.893 (VOO-FVOOT) 0.478 (FVOO-FVOOT) 0.445 (VOO-FVOO) | 0.893 |
| **Triglycerides (mg/dL)** | 7.29 ± 1.19 | 7.25 ± 1.36 | 7.17 ± 1.46 | 0.628 (VOO-FVOOT) 0.749 (FVOO-FVOOT) 0.882 (VOO-FVOO) | 0.628 |
| **ApoA1 (g/L)** | 65 ± 11 | 66 ± 12 | 64 ± 14 | 0.271 (VOO-FVOOT) 0.150 (FVOO-FVOOT) 0.510 (VOO-FVOO) | 0.271 |
| **ApoA2 (g/L)** | 16.91 ± 2.20 | 17.61 ± 3.10 | 16.8 ± 3.38 | 0.818 (VOO-FVOOT) 0.152 (FVOO-FVOOT) 0.158 (VOO-FVOO) | 0.818 |
| **Free cholesterol (mg/dL)** | 12.98 ± 4.47 | 13.61 ± 5.90 | 12.53 ± 5.42 | 0.381 (VOO-FVOOT) 0.107 (FVOO-FVOOT) 0.303 (VOO-FVOO) | 0.381 |
| **Esterified cholesterol (mg/dL)** | 18.18 ± 6.30 | 18.25 ± 5.78 | 18.56 ± 7.36 | 0.624 (VOO-FVOOT) 0.625 (FVOO-FVOOT) 0.909 (VOO-FVOO) | 0.624 |
| **Phospholipids (mg/dL)** | 59.73 ± 16.26 | 61.45 ± 14.89 | 59.49 ± 15.88 | 0.883 (VOO-FVOOT) 0.357 (FVOO-FVOOT) 0.395 (VOO-FVOO) | 0.883 |
| **Free cholesterol / Total cholesterol (AU)** | 0.43 ± 0.06 | 0.42 ± 0.08 | 0.40 ± 0.08 | 0.056 (VOO-FVOOT) 0.063 (FVOO-FVOOT) 0.898 (VOO-FVOO) | 0.056 |
| **Esterified cholesterol / Total cholesterol (AU)** | 0.57 ± 0.06 | 0.58 ± 0.08 | 0.60 ± 0.08 | 0.056 (VOO-FVOOT) 0.063 (FVOO-FVOOT) 0.898 (VOO-FVOO) | 0.056 |
| **Phospholipids / Free cholesterol (AU)** | 4.97 ± 0.81 | 4.99 ± 0.89 | 5.44 ± 1.39 | 0.028 (VOO-FVOOT)* 0.027 (FVOO-FVOOT)* 0.900 (VOO-FVOO) | 0.028 ^$^ |
| **Esterified cholesterol / Free cholesterol (AU)** | 1.53 ± 0.35 | 1.50 ± 0.33 | 1.73 ± 0.56 | 0.029 (VOO-FVOOT)* 0.007 (FVOO-FVOOT)* 0.604 (VOO-FVOO) | 0.029 ^$^ |

Values expressed as post-intervention mean ± S.D. or median (25th to 75th percentile). Post-intervention was adjusted by its pre-intervention. Intra-intervention p-values were not significant. Inter-intervention p-value: * p<0.05; Linear trend p-value: $ p<0.05. HDL, high-density lipoprotein; VOO, virgin olive oil; FVOO, functional virgin olive oil enriched with its own phenolic compounds; FVOOT, functional virgin olive oil enriched with its own phenolic compounds plus additional complementary ones from thyme; Apo, Apolipoprotein; AU, arbitrary units.

**Table S5. Lipid classes, Lipid Class-Fatty Acid combination, and Lipid Species analyzed in the HDL of the VOHF study**.

|  |  |  |  |
| --- | --- | --- | --- |
| **Lipid Classes** | | **LC-FA combination (n)** | **Lipid Species (n)** |
| *Phospholipids* | |  |  |
|  | Lysophosphatidylcholines | 13 | 15 |
|  | Lysophosphatidylethanolamines | 6 | 6 |
|  | Phosphatidylcholines | 19 | 67 |
|  | Phosphatidylethanolamines | 13 | 67 |
| *Sphingolipids* | |  |  |
|  | Ceramides | 9 | 9 |
|  | Dihydroceramides | 6 | 6 |
|  | Hexosylceramides | 7 | 7 |
|  | Lactosylceramides | 8 | 8 |
|  | Sphingomyelins | 12 | 12 |
| *Neutral Lipids* | |  |  |
|  | Cholesteryl Esters | 26 | 26 |
|  | Free Fatty Acids | 26 | 26 |
|  | Diacylglycerides | 19 | 53 |
|  | Triacylglycerides | 21 | 490 |
| ***TOTAL*** | ***13*** | ***185*** | ***792*** |

LC-FA, Lipid Class-Fatty Acid combination.

**Table S6.** Changes (95% CI) in HDL **Lipid Class-Fatty Acid combination composition** (expressed in %) after 3-weeks of sustained intake of Virgin Olive Oil (VOO) and two functional olive oils, one enriched with VOO phenolic content (FVOO) and other with them and phenolic compounds from thyme (FVOOT).

| **Lipid** | **Baseline** | **Changes after Olive Oil Intervention** | | | |  | **Between-intervention differences** | | |
| --- | --- | --- | --- | --- | --- | --- | --- | --- | --- |
|  |  | **VOO** | **FVOO** | **FVOOT** | **P trend** |  | **FVOO vs VOO** | **FVOOT vs VOO** | **FVOO vs FVOOT** |
| CE (FA18:1) | 21.3 ± 2.40 | **0.966** | **1.45** | **0.58** | **<0.001**^†^ |  | 0.278 (-0.51 to 1.1) | -0.395 (-1.2 to 0.40) | -0.673 (-1.5 to 0.15) |
|  |  | **(0.36 to 1.6)** | **(0.63 to 2.28)** | **(0.05 to 1.56)** |  |  | *P* = 0.487 | *P* = 0.324 | *P* = 0.109 |
| CE (FA22:6) | 1.06 ± 0.28 | **-0.175** | **- 0.088** | **-0.120** | **0.005**^†^ |  | **0.105 (0.02 to 0.19)** | 0.056 (-0.03 to 0.14) | -0.049 (-0.14 to 0.04) |
|  |  | **(-0.25 to -0.10)** | **(-0.17 to -0.002)** | **(-0.21 to- 0.03)** |  |  | ***P* = 0.015** | *P* = 0.186 | *P* = 0.272 |
| PC (FA18:1) | 12.2 ± 1.71 | **1.15** | **1.04** | 0.460 | **0.010**^†^ |  | -0.130 (-1.2 to 0.96) | ***-0.990 (-2.1 to 0.15)*** | -0.860 (-2.2 to 0.45) |
|  |  | **(0.23 to 2.1)** | **(0.24 to 1.8)** | (-1.1 to 1.9) |  |  | *P* = 0.812 | ***P = 0.088*** | *P* = 0.195 |
| SM (FA22:1) | 10.1 ± 1.22 | **0.771** | **0.910** | **0.760** | **0.010^*,^**^†^ |  | -0.102 (-0.68 to 0.48) | -0.022 (-0.60 to 0.55) | 0.080 (-0.53 to 0.69) |
|  |  | **(0.26 to 1.3)** | **(0.09 to 1.7)** | **(0.04 to 1.5)** |  |  | *P* = 0.728 | *P* = 0.939 | *P* = 0.795 |
| SM (FA26:0) | 0.046 ± 0.01 | **-0.005** | **-0.005** | -0.003 | **0.009**^†^ |  | 0.000 (-0.003 to 0.004) | 0.002 (-0.001 to 0.006) | 0.002 (-0.002 to 0.006) |
|  |  | **(-0.01 to -0.001)** | **(-0.01 to 0.000)** | (-0.01 to 0.002) |  |  | *P* = 0.822 | *P* = 0.228 | *P* = 0.357 |
| TAG (FA18:1) | 43.0 ± 4.61 | **3.09** | **3.00** | 1 .78 | **<0.001**^†^ |  | -0.069 (-1.7 to 1.5) | ***-1.44 (-3.0 to 0.13)*** | -1.37 (-3.0 to 0.30) |
|  |  | **(1.2 to 4.9)** | **(1.0 to 4.9)** | (-0.37 to 3.9) |  |  | *P* = 0.931 | ***P = 0.073*** | *P* = 0.107 |
| TAG (FA18:2) | 16.6 ± 3.39 | **- 2.20** | **- 2.37** | -1 .27 | **<0.001**^†^ |  | -0.297 (-1.6 to 1.0) | **1.43 (0.14 to 2.7)** | **1.72 (0.35 to 3.1)** |
|  |  | **(-4.0 to -0.40)** | **(-4.0 to -0.72)** | (-3.4 to 0.85) |  |  | *P* = 0.652 | ***P* = 0.030** | ***P* = 0.014** |
| TAG (FA20:1) | 0.209 ± 0.04 | ***0.022*** | 0.012 | 0.011 | ***0.097^†^*** |  | -0.005 (-0.03 to 0.02) | -0.014 (-0.04 to 0.008) | -0.009 (-0.03 to 0.01) |
|  |  | ***(-0.002-0.05)*** | (-0.01-0.04) | (-0.01-0.04) |  |  | *P* = 0.634 | *P* = 0.207 | *P* = 0.463 |

n=33. CE, cholesteryl ester; PC, phosphatidylcholine; SM, sphingomyelin; TAG, triacylglycerols. In the GLM, the P-value for trend among oils VOO, FVOO, FVOOT: * linear trend; † quadratic trend. Between-intervention differences by ANCOVA Model adjusted by age sex, sequence, HDL particle number, and baseline values. Significant values in **bold** and borderline ones in ***italic bold.***

**Table S7**. Changes (95%CI) in HDL **Lipid Species Concentration** (nmol/g) after 3-weeks of sustained intake of Virgin Olive Oil (VOO) and two functional olive oils, one enriched with VOO phenolic content (FVOO) and other with them and phenolic compounds from thyme (FVOOT).

| **Lipid** | **Baseline** | **Changes after Olive Oil Intervention** | | | |  | **Between-intervention differences** | | |
| --- | --- | --- | --- | --- | --- | --- | --- | --- | --- |
|  |  | **VOO** | **FVOO** | **FVOOT** | **P trend** |  | **FVOO vs VOO** | **FVOOT vs VOO** | **FVOO vs FVOOT** |
| PC (FA18:1/FA18:1) | 12.9 ± 6.04 | **4.07** | **4.66** | **3.40** | **<0.001**^†^ |  | 0.343 (-2.2 to 2.9) | 0.253 (-2.2 to 2.7) | -0.090 (-2.7 to 2.6) |
|  |  | **(2.4 to 5.7)** | **(2.3 to 7.1)** | **(0.95 to 5.8)** |  |  | *P* = 0.787 | *P* = 0.841 | *P* = 0.947 |
| TAG 51:4 (FA18:2) | 0.20 ± 0.08 | **-0.043** | **-0.045** | -0.027 | **0.001**^†^ |  | 0.002 (-0.03 to 0.03) | 0.022 (-0.007 to 0.05) | 0.020 (-0.01 to 0.05) |
|  |  | **(-0.07 to -0.02)** | **(-0.07 to -0.02)** | (-0.06 to 0.003) |  |  | *P* = 0.909 | *P* = 0.134 | *P* = 0.192 |
| ^a^TAG 54:7 (FA22:5) | 0.036 ± 0.01 | -0.006 | -0.002 | -0.006 | 0.186^*^ |  | 0.006 (-0.006 to 0.02) | 0.001 (-0.01 to 0.01) | -0.005 (-0.02 to 0.008) |
|  |  | (-0.02 to 0.01) | (-0.02 to 0.01) | (-0.02 to 0.004) |  |  | *P* = 0.307 | P = 0.835 | *P* = 0.459 |
| TAG 56:8 (FA18:2) | 0.29 ± 0.14 | **-0.086** | -0.059 | -0.019 | **0.003**^†^ |  | 0.038 (-0.02 to 0.10) | **0.085 (0.02 to 0.14)** | 0.047 (-0.02 to 0.1) |
|  |  | **(-0.15 to -0.02)** | (-0.14 to 0.02) | (-0.11 to 0.07) |  |  | *P* = 0.222 | ***P* = 0.007** | *P* = 0.150 |

n=33; ^a^ measured in 14 samples; TAG (51:5/18:3) was not analyzed due to the lack of enough data. PC, phosphatidylcholine; TAG, triacylglycerols. In the GLM, the P-value for trend among oils VOO, FVOO, FVOOT: * linear trend; † quadratic trend. Between-intervention differences by ANCOVA Model adjusted by age sex, sequence, HDL particle number, and baseline values. Significant values in **bold**.

**Table S8.** Changes (95%CI) in HDL Diacylglycerols (DAG) and Phosphatidylcholine (PC) Lipid Species Composition (%) after 3 weeks of sustained intake of Virgin Olive Oil (VOO) and two functional olive oils, one enriched with VOO phenolic content (FVOO) and other with them and phenolic compounds from thyme (FVOOT).

| **Lipid** | **Baseline** | **Changes after Olive Oil Intervention** | | | |  | **Between-intervention differences** | | |
| --- | --- | --- | --- | --- | --- | --- | --- | --- | --- |
|  |  | **VOO** | **FVOO** | **FVOOT** | **P trend** |  | **FVOO vs VOO** | **FVOOT vs VOO** | **FVOO vs FVOOT** |
| ^a^DAG (FA18:2/FA22:6) | 0.73 ± 0.43 | -0.100 | -0.117 | 0.384 | **0.010**^†^ |  | -0.007 (-0.44 to 0.43) | **0.538 (0.04 to 1.0)** | **0.545 (0.07 to 1.0)** |
|  |  | (-0.55 to 0.35) | (-0.46 to 0.23) | (-0.40 to 1.2) |  |  | *P* = 0.972 | ***P* = 0.036** | ***P* = 0.025** |
| PC (FA18:1/FA18:1) | 1.04 ± 0.34 | **0.258** | **0.223** | **0.169** | **0.003**^†^ |  | -0.053 (-0.20 to 0.10) | -0.040 (-0.19 to 0.11) | 0.013 (-0.14 to 0.17) |
|  |  | **(0.07 to 0.45)** | **(0.03 to 0.42)** | **(0.009 to 0.33)** | **0.013^*^** |  | *P* = 0.485 | *P* = 0.599 | *P* = 0.867 |

n =33; ^a^ in 12 samples. DAG, diacylglycerols; PC, phosphatidylcholine. In the GLM, P-value for trend among oils VOO, FVOO, FVOOT: * linear trend; † quadratic trend. Between-intervention differences by ANCOVA Model adjusted by age sex, sequence, HDL particle number, and baseline values. Significant values in **bold**.

**Table S9.** Increases (Mean, 95%CI) in HDL Triacylglycerol (TAG) Lipid Species Composition (%) after 3 weeks of Sustained Consumption of Virgin Olive Oil (VOO) and two functional olive oils, one enriched with VOO phenolic content (FVOO) and other with them and phenolic compounds from thyme (FVOOT).

| **Lipid** | **Baseline** | **Changes after Olive Oil Intervention** | | | |  | **Between-intervention differences** | | |
| --- | --- | --- | --- | --- | --- | --- | --- | --- | --- |
|  |  | **VOO** | **FVOO** | **FVOOT** | **P trend** |  | **FVOO vs VOO** | **FVOOT vs VOO** | **FVOO vs FVOOT** |
| TAG 50:3 (FA14:1) | 0.031 ± 0.01 | **0.006** | **0.004** | 0.003 | **0.006**^†^ |  | -0.002 (-0.01 to 0.002) | -0.003 (-0.01 to 0.001) | -0.001 (-0.005 to 0.003) |
|  |  | **(0.003 to 0.01)** | **(0.000 to 0.01)** | (-0.001 to 0.01) |  |  | *P* = 0.405 | P = 0.170 | *P* = 0.619 |
| TAG 52:1 (FA18:1) | 1.43 ± 0.28 | **0.156** | **0.161** | 0.090 | **0.002**^†^ |  | -0.008 (-0.13 to 0.12) | ***-0.114 (-0.24 to 0.009)*** | -0.106 (-0.24 to 0.02) |
|  |  | **(0.02 to 0.29)** | **(0.01 to 0.31)** | (-0.07 to 0.25) |  |  | *P* = 0.896 | ***P = 0.069*** | *P* = 0.112 |
| TAG 52:2 (FA16:0) | 7.55 ± 1.17 | **0.946** | **1.02** | 0.500 | **<0.001**^†^ |  | 0.047 (-0.47 to 0.57) | **-0.619 (-1.1 to -0.11)** | **-0.667 (-1.2 to -0.12)** |
|  |  | **(0.30 to 1.6)** | **(0.39 to 1.6)** | (-0.24 to 1.2) |  |  | *P* = 0.857 | ***P* = 0.019** | ***P* = 0.017** |
| TAG 52:2 (FA18:1) | 13.2 ± 2.29 | **1.80** | **1.82** | 1.02 | **<0.001**^†^ |  | 0.011(-0.95 to 0.97) | **-1.11 (-2.0 to -0.16)** | **1.12 (-2.1 to -0.11)** |
|  |  | **(0.56 to 3.0)** | **(0.63 to 3.0)** | (-0.37 to 2.4) |  |  | *P* = 0.982 | ***P* = 0.022** | ***P* = 0.029** |
| TAG 54:2 (FA16:0) | 0.064 ± 0.01 | **0.008** | 0.006 | 0.005 | **0.032**^†^ |  | -0.001 (-0.01 to 0.006) | -0.004 (-0.01 to 0.003) | -0.003 (-0.01 to 0.004) |
|  |  | **(0.001 to 0.01**) | (-0.001 to 0.01) | (-0.003 to 0.01) |  |  | *P* = 0.723 | *P* = 0.223 | *P* = 0.390 |
| TAG 54:2 (FA18:0) | 0.73 ± 0.16 | **0.078** | 0.087 | 0.044 | **0.010**^†^ |  | 0.005 (-0.07 to 0.08) | -0.047 (-0.12 to 0.03) | -0.052 (-0.13 to 0.02) |
|  |  | **(0.009 to 0.15**) | (-0.03 to 0.20) | (-0.03 to 0.12) |  |  | *P* = 0.888 | *P* = 0.221 | *P* = 0.174 |
| TAG 54:2 (FA18:1) | 1.56 ± 0.38 | **0.231** | **0.235** | 0.125 | **<0.001**^†^ |  | 0.007 (-0.15 to 0.17) | -0.132 (-0.29 to 0.03) | *-****0.140 (-0.30 to 0.02)*** |
|  |  | **(0.07 to 0.39**) | **(0.002 to 0.47**) | (-0.07 to 0.32) |  |  | *P* = 0.927 | *P* = 0.108 | ***P = 0.090*** |
| TAG 54:2 (FA20:1) | 0.064 ± 0.01 | **0.011** | **0.008** | 0.007 | **0.009**^†^ |  | -0.001 (-0.008 to 0.005) | -0.005 (-0.01 to 0.002) | -0.003 (-0.01 to 0.003) |
|  |  | **(0.004 to 0.02**) | **(0.001 to 0.01**) | (-0.001 to 0.01) | **0.038^*^** |  | *P* = 0.645 | *P* = 0.139 | *P* = 0.309 |
| TAG 54:3 (FA18:1) | 6.71 ± 2.54 | **1.28** | **1.24** | 0.721 | **<0.001**^†^ |  | 0.018 (-0.87 to 0.91) | -0.559 (-1.4 to 0.33) | 0.577 (-1.5 to 0.31) |
|  |  | **(0.40 to 2.2**) | **(0.12 to 2.3**) | (-0.36 to 1.8) |  |  | *P* = 0.969 | *P* = 0.213 | *P* = 0.200 |
| TAG 54:3 (FA20:2) | 0.063 ± 0.02 | **0.007** | 0.004 | 0.004 | **0.027**^†^ |  | -0.003 (-0.01 to 0.003) | -0.004 (-0.01 to 0.002) | -0.001 (-0.01 to 0.005) |
|  |  | **(0.001 to 0.01**) | (-0.002 to 0.01) | (-0.004 to 0.01) |  |  | *P* = 0.312 | *P* = 0.201 | *P* = 0.789 |
| TAG 54:4 (FA20:3) | 0.15 ± 0.05 | **0.016** | 0.014 | 0.016 | ***0.070^†^*** |  | -0.003 (-0.02 to 0.01) | -0.003 (-0.02 to 0.01) | 0.000 (-0.02 to 0.01) |
|  |  | **(0.004 to 0.03**) | (-0.003 to 0.03) | (-0.009 to 0.04) |  |  | *P* = 0.741 | *P* = 0.744 | *P* = 0.996 |
| TAG 56:3 (FA18:1) | 0.083 ± 0.02 | **0.013** | 0.012 | 0.011 | **0.034**^†^ |  | 0.001 (-0.01 to 0.01) | -0.004 (-0.01 to 0.007) | -0.005 (-0.02 to 0.006) |
|  |  | **(0.001 to 0.02**) | (-0.001 to 0.03) | (-0.002 to 0.02) | **0.032^*^** |  | *P* = 0.825 | *P* = 0.497 | *P* = 0.370 |
| TAG 56:3 (FA20:1) | 0.039 ± 0.01 | **0.007** | **0.008** | 0.006 | **0.022**^†^ |  | 0.002 (-0.004 to 0.008) | -0.002 (-0.01 to 0.004) | -0.004 (-0.01 to 0.002) |
|  |  | **(0.000 to 0.01**) | **(0.000 to 0.01**) | (-0.002 to 0.01) | **0.048^*^** |  | *P* = 0.548 | *P* = 0.491 | *P* = 0.199 |
| TAG 56:4 (FA20:2) | 0.028 ± 0.01 | **0.005** | 0.003 | 0.004 | **0.041**^†^ |  | -0.001 (-0.005 to 0.002) | -0.002 (-0.006 to 0.002) | 0.000 (-0.004 to 0.003) |
|  |  | **(0.001 to 0.01**) | (-0.002 to 0.01) | (-0.001 to 0.01) | ***0.061*^*^** |  | *P* = 0.486 | *P* = 0.345 | *P* = 0.806 |
| TAG 56:4 (FA20:3) | 0.032 ± 0.01 | **0.003** | **0.005** | 0.003 | **0.028**^†^ |  | 0.002 (-0.001 to 0.005) | -0.001 (-0.004 to 0.002) | -0.002(-0.01 to 0.001) |
|  |  | **(0.000 to 0.005**) | **(0.000 to 0.01**) | (-0.001 to 0.01) | **0.026^*^** |  | *P* = 0.292 | *P* = 0.635 | *P* = 0.128 |
| TAG 56:5 (FA20:3) | 0.081 ± 0.02 | **0.016** | **0.016** | ***0.014*** | **0.003**^†^ |  | -0.001 (-0.01 to 0.01) | -0.004 (-0.02 to 0.008) | -0.003 (-0.01 to 0.01) |
|  |  | **(0.006 to 0.03)** | **(0.001 to 0.03)** | ***(0.000 to 0.03*)** | **0.023^*^** |  | *P* = 0.846 | *P* = 0.495 | *P* = 0.626 |

n=33. TAG, triacylglycerols. In the GLM, P-value for trend among oils VOO, FVOO, FVOOT: * linear trend; † quadratic trend. Between-intervention differences by ANCOVA Model adjusted by age sex, sequence, HDL particle number, and baseline values. Significant values in **bold** and borderline ones in ***italic bold***.

**Table S10.** Decreases (Mean, 95%CI) in HDL Triacylglycerol (TAG) Lipid Species Composition (%) after 3-weeks of Sustained Consumption of Virgin Olive Oil (VOO) and two functional olive oils, one enriched with VOO phenolic content (FVOO) and other with them and phenolic compounds from thyme (FVOOT).

| **Lipid** | **Baseline** | **Changes after Olive Oil Intervention** | | | |  | **Between-intervention differences** | | |
| --- | --- | --- | --- | --- | --- | --- | --- | --- | --- |
|  |  | **VOO** | **FVOO** | **FVOOT** | **P trend** |  | **FVOO vs VOO** | **FVOOT vs VOO** | **FVOO vs FVOOT** |
| TAG 50:4 (FA14:0) | 0.078 ± 0.03 | -0.017 | **-0.021** | -0.009 | **<0.001**^†^ |  | -0.006 (-0.02 to 0.007) | ***0.012 (-0.01 to 0.02)*** | **0.017 (0.004 to 0.03)** |
|  |  | (-0.03 to 0.001) | **(-0.04 to -0.004**) | (-0.03 to 0.01) |  |  | *P* = 0.390 | ***P = 0.062*** | ***P* = 0.011** |
| TAG 50:4 (FA18:2**)** | 0.14 ± 0.05 | **-0.033** | **-0.038** | -0.018 | **<0.001**^†^ |  | -0.006 (-0.02 to 0.01) | ***0.019 (0.000 to 0.04)*** | **0.025 (0.004 to 0.05)** |
|  |  | **(-0.06 to -0.003**) | **(-0.06 to -0.01**) | (-0.05 to 0.01) |  |  | *P* = 0.564 | ***P = 0.055*** | ***P* = 0.019** |
| TAG 51:3 (FA15:0) | 0.069 ± 0.02 | **-0.008** | **-0.008** | -0.004 | **0.006**^†^ |  | 0.001 (-0.005 to 0.01) | **0.006 (0.000 to 0.01)** | 0.005 (-0.001 to 0.01) |
|  |  | **(-0.02 to -0.001**) | **(-0.01 to -0.000**) | (-0.001 to 0.02) |  |  | *P* = 0.702 | ***P* = 0.045** | *P* = 0.127 |
| TAG 51:3 (FA18:2**)** | 0.10 ± 0.02 | **-0.012** | **-0.013** | **-0.010** | **0.007**^†^ |  | -0.001 (-0.01 to 0.008) | 0.002 (-0.007 to 0.01) | 0.004 (-0.006 to 0.01) |
|  |  | **(-0.02 to -0.001**) | **(-0.02 to -0.003**) | **(-0.02 to -0.001**) | **0.013^*^** |  | *P* = 0.760 | *P* = 0.559 | *P* = 0.432 |
| TAG 51:4 (FA15:0) | 0.022 ± 0.01 | **-0.005** | **-0.006** | -0.002 | **<0.001**^†^ |  | -0.000 (-0.003 to 0.003) | **0.003 (0.000 to 0.01)** | **0.003 (0.000 to 0.01)** |
|  |  | **(-0.01 to -0.002**) | **(-0.01 to -0.002**) | (-0.01 to 0.002) |  |  | *P* = 0.951 | ***P* = 0.033** | ***P* = 0.041** |
| TAG 51:4 (FA18:2) | 0.038 ± 0.01 | **-0.009** | **-0.010** | -0.006 | **<0.001**^†^ |  | -0.001 (-0.004 to 0.003) | 0.000 (-0.003 to 0.004) | 0.001 (-0.003 to 0.004) |
|  |  | **(-0.01 to -0.004**) | **(-0.01 to -0.004**) | (-0.01 to 0.001 | **0.031^*^** |  | *P* = 0.717 | *P* = 0.960 | *P* = 0.696 |
| TAG 52:3 (FA16:0) | 6.11 ± 0.89 | **-0.463** | **-0.480** | -0.315 | **0.002**^†^ |  | -0.058 (-0.37 to 0.25) | 0.176 (-0.13 to 0.48) | 0.235 (-0.09 to 0.56) |
|  |  | **(-0.87 to -0.06**) | **(-0.92 to -0.04**) | (-0.78 to 0.15) |  |  | *P* = 0.710 | *P* = 0.256 | *P* = 0.156 |
| TAG 52:4 (FA16:0) | 1.68 ± 0.59 | **-0.362** | **-0.403** | -0.229 | **<0.001**^†^ |  | -0.057 (-0.27 to 0.15) | **0.213 (0.007 to 0.42)** | **0.271 (0.05 to 0.49)** |
|  |  | **(-0.65 to -0.07**) | **(-0.69 to -0.12**) | (-0.58 to 0.12) |  |  | *P* = 0.588 | ***P* = 0.043** | ***P* = 0.016** |
| TAG 52:4 (FA18:2) | 2.86 ± 0.92 | **-0.615** | **-0.635** | -0.394 | **<0.001**^†^ |  | -0.062 (-0.39 to 0.27) | ***0.326 (-0.002 to 0.65)*** | **0.388 (0.06 to 0.72)** |
|  |  | **(-1.1 to -0.13**) | **(-1.1 to -0.18**) | (-0.95 to 0.16) |  |  | *P* = 0.709 | ***P = 0.051*** | ***P* = 0.021** |
| TAG 52:5 (FA16:0) | 0.16 ± 0.08 | **-0.032** | **-0.045** | -0.017 | **0.001**^†^ |  | -0.008 (-0.04 to 0.02) | 0.023 (-0.006 to 0.05) | **0.031 (0.002 to 0.06)** |
|  |  | **(-0.06 to -0.001**) | **(-0.09 to -0.002**) | (-0.07 to 0.04) |  |  | *P* = 0.590 | *P* = 0.119 | ***P* = 0.038** |
| TAG 52:5 (FA18:2) | 0.36 ± 0.14 | **-0.084** | **-0.095** | -0.048 | **<0.001**^†^ |  | -0.013 (-0.06 to 0.04) | **0.055 (0.003 to 0.11)** | **0.068 (0.02 to 0.12)** |
|  |  | **(-0.16 to -0.009**) | **(-0.17 to -0.02**) | (-0.14 to 0.04) |  |  | *P* = 0.634 | ***P* = 0.037** | ***P* = 0.011** |
| TAG 52:5 (FA18:3) | 0.24 ± 0.09 | **-0.039** | **-0.052** | -0.020 | **<0.001**^†^ |  | -0.009 (-0.04 to 0.03) | ***0.030 (-0.005 to 0.06)*** | **0.039 (0.004 to 0.07)** |
|  |  | **(-0.08 to 0.000**) | **(-0.10 to -0.004**) | (-0.09 to 0.05) |  |  | *P* = 0.629 | ***P = 0.089*** | ***P* = 0.030** |
| ^a^TAG 52:6 (FA16:0) | 0.038 ± 0.01 | -0.003 | -0.003 | -0.001 | **0.032**^†^ |  | 0.001 (-0.002 to 0.005) | 0.003 (-0.001 to 0.006) | 0.001 (-0.002 to 0.005) |
|  |  | (-0.006 to 0.001) | (-0.008 to 0.002) | (-0.006 to 0.005) |  |  | *P* = 0.450 | P = 0.107 | *P* = 0.387 |
| TAG 52:6 (FA16:1) | 0.026 ± 0.01 | **-0.007** | **-0.007** | -0.003 | **0.002**^†^ |  | 0.001 (-0.003 to 0.005) | **0.004 (0.000 to 0.009)** | 0.003 (-0.001 to 0.008) |
|  |  | **(-0.01 to -0.001**) | **(-0.01 to -0.001**) | (-0.01 to 0.006) |  |  | *P* = 0.658 | ***P* = 0.041** | *P* = 0.112 |
| TAG 52:6 (FA18:2) | 0.031 ± 0.01 | -0.008 | **-0.009** | -0.005 | **0.001**^†^ |  | 0.001 (-0.006 to 0.005) | **-0.006 (0.001 to 0.01)** | **0.006 (0.001 to 0.01)** |
|  |  | (-0.02 to 0.000) | **(-0.02 to -0.002**) | (-0.01 to 0.005) |  |  | *P* = 0.844 | ***P* = 0.026** | ***P* = 0.016** |
| TAG 53:3 (FA18:2) | 0.10 ± 0.02 | **-0.012** | **-0.017** | **-0.011** | **<0.001**^†^ |  | -0.005 (-0.01 to 0.002) | 0.003 (-0.004 to 0.009) | **0.007 (0.001 to 0.01)** |
|  |  | **(-0.02 to -0.003**) | **(-0.03 to -0.007**) | **(-0.02 to -0.003**) | **0.001^*^** |  | *P* = 0.161 | *P* = 0.468 | ***P* = 0.035** |
| TAG 54:5 (FA18:0) | 0.024 ± 0.01 | -0.004 | -0.006 | -0.001 | **0.007**^†^ |  | -0.001 (-0.005 to 0.004) | ***0.004 (0.000 to 0.008)*** | **0.005 (0.000 to 0.009)** |
|  |  | (-0.01 to 0.001) | (-0.01 to 0.001) | (-0.01 to 0.009) |  |  | *P* = 0.776 | ***P = 0.072*** | ***P* = 0.038** |
| TAG 54:6 (FA16:0) | 0.090 ± 0.05 | -0.022 | **-0.017** | -0.009 | **0.001**^†^ |  | 0.009 (-0.004 to 0.02) | **0.013 (0.000 to 0.03)** | 0.004 (-0.008 to 0.02) |
|  |  | (-0.04 to 0.01) | **(-0.03 to -0.000**) | (-0.03 to 0.02) |  |  | *P* = 0.178 | ***P* = 0.045** | *P* = 0.505 |
| TAG 54:6 (FA18:2) | 0.43 ± 0.27 | -0.143 | **-0.171** | -0.068 | **<0.001**^†^ |  | -0.028 (-0.12 to 0.07) | **0.114 (0.02 to 0.21)** | **0.142 (0.04 to 0.24)** |
|  |  | (-0.29 to 0.005) | **(-0.32 to -0.03**) | (-0.27 to 0.14) |  |  | *P* = 0.573 | ***P* = 0.022** | ***P* = 0.005** |
| TAG 54:6 (FA20:4) | 0.22 ± 0.08 | **-0.036** | **-0.040** | -0.019 | **<0.001**^†^ |  | -0.004 (-0.02 to 0.02) | 0.017 (-0.004 to 0.04) | ***0.021 (-0.001 to 0.04)*** |
|  |  | **(-0.06 to -0.01**) | **(-0.07 to -0.01**) | (-0.05 to 0.02) |  |  | *P* = 0.740 | *P* = 0.113 | ***P = 0.056*** |
| TAG 54:7 (FA18:2) | 0.073 ± 0.06 | -0.029 | **-0.034** | -0.008 | **0.003**^†^ |  | -0.003 (-0.03 to 0.02) | **0.027 (0.003 to 0.05)** | **0.030 (0.006 to 0.05)** |
|  |  | (-0.06 to 0.004) | **(-0.07to -0.002**) | (-0.06 to 0.04) |  |  | *P* = 0.799 | ***P* = 0.028** | ***P* = 0.015** |
| TAG 54:7 (FA20:4) | 0.031 ± 0.01 | **-0.006** | **-0.006** | -0.002 | **<0.001**^†^ |  | 0.000 (-0.004 to 0.004) | **0.004 (0.000 to 0.008)** | ***0.004 (0.000 to 0.008)*** |
|  |  | **(-0.01 to -0.001**) | **(-0.01 to -0.000**) | (-0.01 to 0.005) |  |  | *P* = 0.851 | ***P* = 0.044** | ***P = 0.068*** |
| ^b^TAG 54:8 (FA18:2) | 0.011 ± 0.01 | **-0.005** | -0.005 | -0.001 | **0.024**^†^ |  | 0.000 (-0.004 to 0.003) | **0.004 (0.000 to 0.007)** | **0.004 (0.001 to 0.008)** |
|  |  | **(-0.01 to -0.000**) | (-0.01 to 0.002) | (-0.01 to 0.01) |  |  | *P* = 0.799 | ***P* = 0.034** | ***P* = 0.019** |
| TAG 55:1 (FA16:0) | 0.041 ± 0.03 | -0.015 | -0.009 | -0.006 | **0.010**^†^ |  | 0.008 (-0.003 to 0.02) | ***0.009 (-0.001 to 0.02)*** | 0.002 (-0.01 to 0.01) |
|  |  | (-0.04 to 0.007) | (-0.02 to 0.001) | (-0.03 to 0.02) |  |  | *P* = 0.140 | ***P = 0.077*** | *P* = 0.760 |
| TAG 56:7 (FA18:2) | 0.079 ± 0.02 | **-0.015** | **-0.013** | -0.005 | **<0.001**^†^ |  | 0.002 (-0.007 to 0.01) | **0.011 (0.002 to 0.02)** | 0.009 (-0.001 to 0.01) |
|  |  | **(-0.03 to -0.003)** | **(-0.01 to -0.001)** | (-0.02 to 0.01) |  |  | *P* = 0.626 | ***P* = 0.023** | ***P = 0.072*** |
| TAG 56:7 (FA22:5) | 0.069 ± 0.03 | -0.016 | -0.012 | -0.007 | **0.011**^†^ |  | 0.007 (-0.004 to 0.02) | ***0.010 (-0.001 to 0.02)*** | 0.003 (-0.007 to 0.01) |
|  |  | (-0.03 to 0.002) | (-0.03 to 0.001) | (-0.02 to 0.01) |  |  | *P* = 0.205 | ***P = 0.064*** | *P* = 0.556 |
| TAG 56:8 (FA16:0) | 0.043 ± 0.04 | -0.015 | -0.009 | -0.006 | **0.013**^†^ |  | 0.009 (-0.002 to 0.02) | ***0.010 (-0.001 to 0.02)*** | 0.001 (-0.01 to 0.01) |
|  |  | (-0.04 to 0.005) | (-0.02 to 0.002) | (-0.03 to 0.002) |  |  | *P* = 0.105 | ***P = 0.068*** | *P* = 0.840 |
| TAG 56:8 (FA18:2) | 0.061 ± 0.04 | **-0.022** | **-0.016** | -0.007 | **0.001**^†^ |  | 0.008 (-0.005 to 0.02) | **0.016 (0.003 to 0.03)** | 0.008 (-0.005 to 0.02) |
|  |  | **(-0.04 to -0.006)** | **(-0.03 to -0.006)** | (-0.03 to 0.01) |  |  | *P* = 0.204 | ***P* = 0.016** | *P* = 0.241 |
| TAG 56:8 (FA22:6) | -0.77 ± 0.28 | **-0.154** | -0.056 | -0.030 | **0.012**^†^ |  | **0.123 (0.007 to 0.24)** | **0.131 (0.02 to 0.25)** | 0.008 (-0.11 to 0.12) |
| (*log)* |  | **(-0.25 to -0.05)** | (-0.13 to 0.002) | (-0.15 to 0.09) |  |  | ***P* = 0.037** | ***P* = 0.026** | *P* = 0.887 |

n=33;^a^ in 11 samples; ^b^ in 22 samples. TAG 50:5 (FA16:0), TAG 51:5 (FA18:3), TAG 54:7(FA22:5) were not analyzed due to the lack of enough data. In the GLM, P-value for trend among oils VOO, FVOO, FVOOT: * linear trend; † quadratic trend. Between-intervention differences by ANCOVA Model adjusted by age sex, sequence, HDL particle number, and baseline values. Significant values in **bold** and borderline ones in ***italic bold.***

**Table S11. Pearson’s correlation coefficients (R) of the 3-week changes in HDL ChE capacity and those of HDL LC-FA combination composition (%) after 3 weeks of sustained consumption of virgin olive oils.**

|  |  |  |  |  |  |  |
| --- | --- | --- | --- | --- | --- | --- |
| **Variable (3-week changes)** | **VOO (n=30)** | | **FVOO (n=31)** | | **FVOOT (n=30)** | |
|  | **R** | **P** | **R** | **P** | **R** | **P** |
| CE (FA18:1) | -0.242 | 0.198 | 0.173 | 0.360 | ^c^-0.295 | 0.113 |
| CE (FA22:6) | 0.038 | 0.843 | -0.019 | 0.921 | 0.037 | 0.847 |
| PC (FA18:1) | -0.148 | 0.436 | 0.035 | 0.856 | 0.100 | 0.600 |
| SM (FA22:1) | -0.213 | 0.259 | -0.009 | 0.963 | 0.067 | 0.725 |
| SM (FA26:0) | 0.200 | 0.288 | -0.154 | 0.416 | 0.179 | 0.354 |
| TAG (FA18:1) | -0.274 | 0.143 | 0.062 | 0.743 | 0.217 | 0.250 |
| TAG (FA18:2) | **0.365** | **0.047** | **^b^-**0.189 | 0.318 | 0.041 | 0.828 |
| TAG (FA20:1) | -0.225 | 0.232 | 0.017 | 0.928 | -0.011 | 0.955 |

Significant values in **bold**. ^a^ Spearman’s R=-0.424, P=0.018; ^b^ Spearman’s R=-0.363, P=0.049; ^c^ Spearman’s R=0.369, P=0.045.

**Table S12. Pearson’s correlation coefficients (R) of the 3-week changes in HDL ChE capacity and those of HDL Lipid Species Concentration (nmol/g) after 3 weeks of sustained consumption of virgin olive oils.**

|  |  | |  |  |  |  |  |
| --- | --- | --- | --- | --- | --- | --- | --- |
| **Variable (3-week changes)** | **VOO (n= 30)** | | | **FVOO (n=31)** | | **FVOOT (n=30)** | |
|  | **R** | | **P** | **R** | **P** | **R** | **P** |
| PC (FA18:1/FA18:1) | | -0.157 | 0.409 | 0.199 | 0.292 | 0.113 | 0.559 |
| TAG 51:4 (FA18:2) | | 0.143 | 0.452 | 0.187 | 0.323 | -0.016 | 0.932 |
| ^a^TAG 54:7 (FA22:5) | | 0.260 | 0.369 | 0.404 | 0.135 | -0.273 | 0.366 |
| TAG 56:8 (FA18:2) | | ^b^0.225 | 0.233 | 0.022 | 0.907 | 0.074 | 0.696 |

^a^ Only 15 samples available. ^b^ Spearman´s R=0.394, P=0.03.

**Table S13. Pearson’s correlation coefficients (R) of the 3-week changes in HDL ChE capacity and those of HDL Lipid Species Composition (%) of DAG (FA18:2/FA22:6) and PC (FA18:1/FA18:1) after 3 weeks of sustained consumption of virgin olive oils.**

|  |  |  |  |  |  |  |
| --- | --- | --- | --- | --- | --- | --- |
| **Variable (3-week changes)** | **VOO (n= 30)** | | **FVOO (n=31)** | | **FVOOT (n=30)** | |
|  | **R** | **P** | **R** | **P** | **R** | **P** |
| ^a^ DAG (FA18:2/FA22:6) | 0.378 | 0.356 | -0.011 | 0.971 | -0.314 | 0.345 |
| PC (FA18:1/FA18:1) | -0.122 | 0.520 | 0.120 | 0.528 | 0.416 | 0.025 |

^a^n=8 for VOO, 14 for FVOO, and 11 for FVOOT

**Table S14. Pearson’s correlation coefficients (R) of the 3-week changes in HDL ChE and those of Triacylglycerol Lipid Species Composition (%) in HDL which after 3 weeks of sustained consumption of virgin olive oils.**

|  |  |  |  |  |  |  |
| --- | --- | --- | --- | --- | --- | --- |
| **Variable (3-week changes)** | **VOO (n= 30)** | | **FVOO (n=31)** | | **FVOOT (n=30)** | |
|  | **R** | **P** | **R** | **P** | **R** | **P** |
| TAG 50:3 (FA14:1) | **-0.547** | **0.002** | 0.192 | 0.310 | -0.190 | 0.313 |
| TAG 52:1 (FA18:1) | -0.339 | ***0.067*** | 0.152 | 0.421 | 0.013 | 0.945 |
| TAG 52:2 (FA16:0) | -0.322 | ***0.082*** | 0.113 | 0.551 | 0.122 | 0.522 |
| TAG 52:2 (FA18:1) | -0.358 | ***0.052*** | 0.096 | 0.615 | 0.102 | 0.593 |
| TAG 54:2 (FA16:0) | -0.328 | ***0.077*** | 0.227 | 0.227 | 0.168 | 0.374 |
| TAG 54:2 (FA18:0) | -0.279 | 0.136 | 0.032 | 0.865 | 0.128 | 0.499 |
| TAG 54:2 (FA18:1) | -0.272 | 0.146 | 0.027 | 0.887 | 0.159 | 0.402 |
| TAG 54:2 (FA20:1) | -0.339 | ***0.066*** | 0.174 | 0.357 | 0.079 | 0.680 |
| TAG 54:3 (FA18:1) | -0.082 | 0.668 | -0.007 | 0.971 | 0.293 | 0.116 |
| TAG 54:3 (FA20:2) | -0.161 | 0.396 | 0.244 | 0.194 | -0.012 | 0.951 |
| TAG 54:4 (FA20:3) | -0.166 | 0.379 | 0.030 | 0.875 | -0.052 | 0.783 |
| TAG 56:3 (FA18:1) | -0.255 | 0.173 | 0.024 | 0.899 | 0.257 | 0.170 |
| TAG 56:3 (FA20:1) | -0.256 | 0.172 | 0.054 | 0.775 | 0.324 | ***0.080*** |
| TAG 56:4 (FA20:2) | -0.147 | 0.439 | 0.085 | 0.656 | 0.105 | 0.581 |
| TAG 56:4 (FA20:3) | -0.210 | 0.264 | -0.179 | 0.343 | 0.087 | 0.648 |
| TAG 56:5 (FA20:3) | -0.101 | 0.594 | -0.023 | 0.903 | 0.082 | 0.668 |

Significant values in **bold** and borderline ones in ***italic bold***.

**Table S15. Pearson’s correlation coefficients (R) of the 3-week changes in HDL ChE and those of Triacylglycerol Lipid Species Composition (%) in HDL which decreased after 3 weeks of sustained consumption of virgin olive oils.**

|  |  |  |  |  |  |  |
| --- | --- | --- | --- | --- | --- | --- |
| **Variable (3-week changes)** | **VOO (n= 30)** | | **FVOO (n=31)** | | **FVOOT (n=30)** | |
|  | **R** | **P** | **R** | **P** | **R** | **P** |
| TAG 50:4 (FA14:0) | 0.305 | 0.101 | -0.024 | 0.879 | -0.141 | 0.457 |
| TAG 50:4 (FA18:2) | 0.280 | 0.135 | -0.111 | 0.558 | -0.180 | 0.340 |
| TAG 51:3 (FA15:0) | **-0.539** | **0.002** | 0.000 | 0.999 | -0.047 | 0.803 |
| TAG 51:3 (FA18:2) | **-0.424** | **0.020** | 0.022 | 0.909 | -0.201 | 0.288 |
| TAG 51:4 (FA15:0) | -0.206 | 0.272 | -0.099 | 0.608 | -0.155 | 0.414 |
| TAG 51:4 (FA18:2) | -0.158 | 0.403 | -0.101 | 0.595 | -0.083 | 0.664 |
| TAG 52:3 (FA16:0) | 0.281 | 0.133 | -0.192 | 0.309 | 0.139 | 0.464 |
| TAG 52:4 (FA16:0) | 0.321 | ***0.083*** | -0.174 | 0.359 | 0.038 | 0.841 |
| TAG 52:4 (FA18:2) | 0.344 | ***0.063*** | ^a^-0.201 | 0.288 | 0.077 | 0.687 |
| TAG 52:5 (FA16:0) | 0.134 | 0.482 | -0.128 | 0.501 | -0.091 | 0.634 |
| TAG 52:5 (FA18:2) | 0.256 | 0.171 | -0.205 | 0.282 | -0.026 | 0.893 |
| TAG 52:5 (FA18:3) | 0.211 | 0.263 | ^b^-0.178 | 0.346 | -0.104 | 0.584 |
| TAG 52:6 (FA16:0) | -0.091 | 0.650 | 0.045 | 0.824 | -0.143 | 0.476 |
| TAG 52:6 (FA16:1) | 0.048 | 0.802 | -0.096 | 0.620 | -0.111 | 0.559 |
| TAG 52:6 (FA18:2) | 0.182 | ***0.093*** | -0.131 | 0.490 | -0.106 | 0.584 |
| TAG 53:3 (FA18:2) | -0.348 | ***0.060*** | -0.092 | 0.629 | -0.091 | 0.631 |
| TAG 54:5 (FA18:0) | 0.063 | 0.740 | -0.138 | 0.466 | -0.047 | 0.810 |
| TAG 54:6 (FA16:0) | 0.050 | 0.792 | -0.047 | 0.804 | -0.064 | 0.739 |
| TAG 54:6 (FA18:2) | 0.134 | 0.481 | ^c^ -0.206 | 0.275 | 0.044 | 0.818 |
| TAG 54:6 (FA20:4) | 0.268 | 0.152 | -0.265 | 0.158 | -0.118 | 0.536 |
| TAG 54:7 (FA18:2) | 0.065 | 0.733 | -0.164 | 0.383 | -0.013 | 0.947 |
| TAG 54:7 (FA20:4) | 0.005 | 0.981 | -0.158 | 0.406 | -0.089 | 0.641 |
| TAG 54:8 (FA18:2) | -0.137 | 0.544 | 0.080 | 0.731 | 0.085 | 0.720 |
| TAG 55:1 (FA16:0) | -0.041 | 0.829 | 0.044 | 0.817 | -0.017 | 0.932 |
| TAG 56:7 (FA18:2) | 0.138 | 0.467 | -0.165 | 0.383 | 0.096 | 0.615 |
| TAG 56:7 (FA22:5) | 0.003 | 0.986 | -0.098 | 0.605 | 0.005 | 0.979 |
| TAG 56:8 (FA16:0) | -0.053 | 0.775 | 0.043 | 0.821 | -0.029 | 0.882 |
| TAG 56:8 (FA18:2) | 0.036 | 0.850 | -0.096 | 0.613 | 0.010 | 0.956 |
| TAG 56:8 (FA22:6) (log) | -0.062 | 0.744 | 0.050 | 0.744 | -0.026 | 0.893 |

Significant values in **bold** and borderline ones in ***italic bold***. ^a^ Spearman´s R= -0.364, P=0.048; ^b^ Spearman´s R= -0.505, P=0.039; ^c^ Spearman´s R= -0.369, P=0.045.

**Table S16. Pearson’s correlation coefficients (R) of the 3-week changes in HDL resistance to oxidation and those of HDL LC-FA combination composition (%) after 3 weeks of sustained consumption of virgin olive oils.**

|  |  |  |  |  |  |  |
| --- | --- | --- | --- | --- | --- | --- |
| **Variable (3-week changes)** | **VOO (n=30)** | | **FVOO (n=31)** | | **FVOOT (n=30)** | |
|  | **R** | **P** | **R** | **P** | **R** | **P** |
| CE (FA18:1) | **0.461** | **0.036** | 0.442 | ***0.099*** | -0.265 | 0.222 |
| CE (FA22:6) | 0.116 | 0.844 | 0.275 | 0.322 | -0.013 | 0.953 |
| PC (FA18:1) | 0.063 | 0.785 | 0.503 | ***0.055*** | -0.223 | 0.307 |
| SM (FA22:1) | **0.482** | **0.027** | 0.273 | 0.325 | -0.028 | 0.901 |
| SM (FA26:0) | 0.155 | 0.502 | -0.031 | 0.912 | -0.210 | 0.434 |
| TAG (FA18:1) | 0.371 | 0.***097*** | 0.251 | 0.367 | **-0.419** | **0.047** |
| TAG (FA18:2) | -0.277 | 0.224 | **-0.703** | **0.003** | **0.445** | **0.034** |
| TAG (FA20:1) | 0.206 | 0.369 | -0.030 | 0.912 | -0.253 | 0.243 |
| Pearson’ s correlation. Significant values in **bold** and borderline ones in ***italic bold***. | | | | | | |

**Table S17. Pearson’s correlation coefficients (R) of the 3-week changes in HDL resistance to oxidation and those of HDL Lipid Species Concentration (nmol/g) after 3 weeks of sustained consumption of virgin olive oils.**

| **Variable (3-week changes)** | **VOO (n= 21)** | | **FVOO (n=15)** | | **FVOOT (n=22)** | |
| --- | --- | --- | --- | --- | --- | --- |
|  | **R** | **P** | **R** | **P** | **R** | **P** |
| PC (FA18:1/FA18:1) | 0.104 | 0.654 | 0.187 | 0.504 | -0.183 | 0.414 |
| TAG 51:4 (FA18:2) | ^b^0.133 | 0.925 | ^c^-0.259 | 0.351 | 0.121 | 0.581 |
| ^a^TAG 54:7 (FA22:5) | -0.402 | 0.324 | -0.207 | 0.694 | 0.489 | 0.107 |
| TAG 56:8 (FA18:2) | -0.195 | 0.398 | -0.137 | 0.625 | 0.303 | 0.160 |

^a^ Only 6 samples for each oil available. ^b^ Spearman´s R=0.372, P=0.097; ^c^ Spearman´s R=-0.529, P=0.045.

**Table S18. Pearson’s correlation coefficients (R) of the 3-week changes in HDL resistance to oxidation and those of HDL Lipid Species Composition (%) of DAG (FA18:2/FA22:6) and PC (FA18:1/FA18:1) after 3 weeks of sustained consumption of virgin olive oils.**

|  |  |  |  |  |  |  |
| --- | --- | --- | --- | --- | --- | --- |
| **Variable (3-week changes)** | **VOO (n= 21)** | | **FVOO (n=15)** | | **FVOOT (n=22)** | |
|  | **R** | **P** | **R** | **P** | **R** | **P** |
| ^a^ DAG (FA18:2/FA22:6) | -0.107 | 0.893 | -0.222 | 0.673 | 0.042 | 0.908 |
| PC (FA18:1/FA18:1) | 0.079 | 0.732 | 0.082 | 0.772 | -0.299 | 0.256 |
|  |  |  |  |  |  |  |
| ^a^(n=12) |  |  |  |  |  |  |

**Table S19. Pearson’s correlation coefficients (R) of the 3-week changes in HDL resistance to oxidation and those of Triacylglycerol Lipid Species Composition (%) in HDL which increased after 3 weeks of sustained consumption of virgin olive oils.**

|  |  |  |  |  |  |  |
| --- | --- | --- | --- | --- | --- | --- |
| **Variable (3-week changes)** | **VOO (n= 21)** | | **FVOO (n=15)** | | **FVOOT (n=22)** | |
|  | **R** | **P** | **R** | **P** | **R** | **P** |
| TAG 50:3 (FA14:1) | 0.127 | 0.585 | 0.167 | 0.551 | **-0.490** | **0.018** |
| TAG 52:1 (FA18:1) | 0.167 | 0.470 | 0.481 | ***0.069*** | -0.374 | ***0.079*** |
| TAG 52:2 (FA16:0) | 0.208 | 0.366 | 0.438 | 0.103 | **-0.438** | **0.037** |
| TAG 52:2 (FA18:1) | 0.232 | 0.312 | 0.441 | 0.100 | **-0.452** | **0.030** |
| TAG 54:2 (FA16:0) | 0.125 | 0.589 | 0.028 | 0.920 | -0.135 | 0.539 |
| TAG 54:2 (FA18:0) | 0.149 | 0.520 | -0.045 | 0.873 | -0.347 | 0.105 |
| TAG 54:2 (FA18:1) | 0.214 | 0.351 | 0.049 | 0.862 | **-0.454** | **0.039** |
| TAG 54:2 (FA20:1) | 0.123 | 0.594 | 0.372 | 0.172 | -0.246 | 0.257 |
| TAG 54:3 (FA18:1) | 0.260 | 0.254 | 0.071 | 0.801 | -0.372 | ***0.081*** |
| TAG 54:3 (FA20:2) | 0.030 | 0.897 | 0.138 | 0.623 | -0.159 | 0.468 |
| TAG 54:4 (FA20:3) | 0.190 | 0.409 | 0.377 | 0.166 | -0.099 | 0.653 |
| TAG 56:3 (FA18:1) | 0.086 | 0.678 | -0.168 | 0.550 | -0.377 | ***0.077*** |
| TAG 56:3 (FA20:1) | 0.245 | 0.285 | -0.063 | 0.198 | -0.344 | 0.108 |
| TAG 56:4 (FA20:2) | 0.126 | 0.587 | 0.022 | 0.938 | -0.046 | 0.836 |
| TAG 56:4 (FA20:3) | 0.134 | 0.563 | -0.149 | 0.596 | 0.142 | 0.517 |
| TAG 56:5 (FA20:3) | 0.249 | 0.276 | -0.005 | 0.986 | -0.029 | 0.895 |

Significant values in **bold** and borderline ones in ***italic bold***.

**Table S20. Pearson’s correlation coefficients (R) of the 3-week changes in HDL resistance to oxidation and those of Triacylglycerol Lipid Species Composition (%) in HDL which decreased after 3 weeks of sustained consumption of virgin olive oils.**

|  |  |  |  |  |  |  |
| --- | --- | --- | --- | --- | --- | --- |
| **Variable (3-week changes)** | **VOO (n= 21)** | | **FVOO (n=15)** | | **FVOOT (n=22)** | |
|  | **R** | **P** | **R** | **P** | **R** | **P** |
| TAG 50:4 (FA14:0) | -0.275 | 0.227 | **-0.528** | **0.043** | 0.346 | 0.106 |
| TAG 50:4 (FA18:2) | -0.333 | 0.140 | **-0.561** | **0.030** | 0.340 | 0.113 |
| TAG 51:3 (FA15:0) | -0.059 | 0.799 | -0.306 | 0.267 | -0.199 | 0.362 |
| TAG 51:3 (FA18:2) | 0.114 | 0.622 | -0.276 | 0.320 | ^b^ 0.210 | 0.336 |
| TAG 51:4 (FA15:0) | -0.238 | 0.299 | 0.400 | 0.139 | 0.226 | 0.299 |
| TAG 51:4 (FA18:2) | -0.115 | 0.618 | -0.454 | ***0.089*** | 0.166 | 0.449 |
| TAG 52:3 (FA16:0) | **-0.633** | **0.002** | **-0.658** | **0.008** | 0.329 | 0.126 |
| TAG 52:4 (FA16:0) | -0.430 | ***0.052*** | **-0.730** | **0.002** | 0.379 | ***0.075*** |
| TAG 52:4 (FA18:2) | -0.395 | ***0.077*** | **-0.789** | **<0.001** | 0.396 | ***0.061*** |
| TAG 52:5 (FA16:0) | -0.221 | 0.336 | -0.198 | 0.478 | 0.179 | 0.415 |
| TAG 52:5 (FA18:2) | -0.137 | 0.555 | **-0.518** | **0.048** | 0.381 | ***0.073*** |
| TAG 52:5 (FA18:3) | -0.242 | 0.290 | -0.355 | 0.194 | 0.204 | 0.351 |
| TAG 52:6 (FA16:0) | 0.019 | 0.940 | -0.027 | 0.933 | 0.166 | 0.473 |
| TAG 52:6 (FA16:1) | 0.039 | 0.867 | -0.128 | 0.662 | 0.320 | 0.137 |
| TAG 52:6 (FA18:2) | 0.014 | 0.951 | -0.315 | 0.252 | 0.416 | ***0.054*** |
| TAG 53:3 (FA18:2) | -0.085 | 0.714 | -0.500 | ***0.058*** | -0.041 | 0.853 |
| TAG 54:5 (FA18:0) | -0.109 | 0.638 | -0.223 | 0.425 | 0.225 | 0.314 |
| TAG 54:6 (FA16:0) | -0.369 | 0.100 | -0.099 | 0.725 | 0.242 | 0.266 |
| TAG 54:6 (FA18:2) | -0.031 | 0.894 | ^d^-0.394 | 0.146 | 0.365 | ***0.085*** |
| TAG 54:6 (FA20:4) | -0.427 | ***0.054*** | -0.321 | 0.243 | 0.180 | 0.412 |
| TAG 54:7 (FA18:2) | 0.001 | 0.997 | -0.145 | 0.607 | 0.248 | 0.253 |
| TAG 54:7 (FA20:4) | -0.208 | 0.366 | -0.238 | 0.392 | 0.138 | 0.529 |
| TAG 54:8 (FA18:2) | -0.088 | 0.766 | -0.066 | 0.866 | -0.292 | 0.240 |
| TAG 55:1 (FA16:0) | -0.280 | 0.219 | -0.190 | 0.499 | ^e^ 0.318 | 0.149 |
| TAG 56:7 (FA18:2) | -0.308 | 0.175 | -0.427 | 0.112 | 0.346 | 0.105 |
| TAG 56:7 (FA22:5) | -0.360 | 0.109 | -0.168 | 0.549 | 0.275 | 0.226 |
| TAG 56:8 (FA16:0) | -0.078 | 0.222 | -0.121 | 0.667 | ^f^0.291 | 0.179 |
| TAG 56:8 (FA18:2) | -0.288 | 0.205 | -0.196 | 0.483 | 0.286 | 0.185 |
| TAG 56:8 (FA22:6) (log) | -0.200 | 0.385 | -0.126 | 0.655 | 0.311 | 0.149 |

^a^ Spearman´s R= -0.302, P=0.030; ^b^ Spearman´s R= -0.447, P=0.033; ^c^ Spearman´s R= -0.232, P=0.059; ^d^ Spearman´s R= -0.450, P=0.092; ^e^ Spearman´s R= 0.403, P=0.063; ^f^ Spearman´s R= 0.353, P=0.099. Significant values in **bold** and borderline ones in ***italic bold***.

***REFERENCES***

[1] A. Pedret, S. Fernández-Castillejo, R.-M. Valls, Ú. Catalán, L. Rubió, M. Romeu, A. Macià, M. C. López de Las Hazas, M. Farràs, M. Giralt, J. I. Mosele, S. Martín-Peláez, A. T. Remaley, M.-I. Covas, M. Fitó, M.-J. Motilva, R. Solà, *Mol. Nutr. Food Res.* **2018**, e1800456.

[2] V. I. Zannis, P. Fotakis, G. Koukos, D. Kardassis, C. Ehnholm, M. Jauhiainen, A. Chroni, *HDL Biogenesis, Remodeling, and Catabolism*, **2015**.

[3] C. J. Fielding, R. J. Havel, K. M. Todd, K. E. Yeo, M. C. Schloetter, V. Weinberg, P. H. Frost, *J. Clin. Invest.* **1995**, *95*, 611–8.

[4] A. Pedret, Ú. Catalán, S. Fernández-Castillejo, M. Farràs, R.-M. Valls, L. Rubió, N. Canela, G. Aragonés, M. Romeu, O. Castañer, R. de la Torre, M.-I. Covas, M. Fitó, M.-J. Motilva, R. Solà, *PLoS One* **2015**, *10*, e0129160.

[5] S. Fernández-Castillejo, L. Rubió, Á. Hernáez, Ú. Catalán, A. Pedret, R.-M. Valls, J. I. Mosele, M.-I. Covas, A. T. Remaley, O. Castañer, M.-J. Motilva, R. Solá, *Mol. Nutr. Food Res.* **2017**, *61*, DOI 10.1002/mnfr.201700445.

[6] S. Fernández-Castillejo, R.-M. Valls, O. Castañer, L. Rubió, Ú. Catalán, A. Pedret, A. Macià, M. L. Sampson, M.-I. Covas, M. Fitó, M.-J. Motilva, A. T. Remaley, R. Solà, Ú. Catalan, A. Pedret, A. Macià, M. L. Sampson, M.-I. Covas, M. Fitó, M.-J. Motilva, A. T. Remaley, R. Solà, *Mol. Nutr. Food Res.* **2016**, *60*, 1544–54.
